# Supplementary material for: Genetic variations and recurrence in stage III Korean colorectal cancer: Insights from tumor-only mutation analysis
Source: PLoS One. 2025 May 23;20(5):e0323302. doi: 10.1371/journal.pone.0323302 (PMC12101642; doi:10.1371/journal.pone.0323302)
Supplement: S1 File — (DOCX) [file pone.0323302.s008.docx]

Table A in S1 File. Summary of mutation profile.

| **Sample Group** | **# of samples** | **Splicing** | **Frameshift indel** | **Nonframeshift indel** | **Nonsense** | **Missense** | **Total** | **Mutations/Mb** |
| --- | --- | --- | --- | --- | --- | --- | --- | --- |
| Overall | 173 | 1471 | 7649 | 3523 | 3631 | 77178 | 93452 | 1544.5 |
| Recurrent | 86 | 24.5 | 53.9 | 70.0 | 24.3 | 882.7 | 1055.3 | 17.4 |
| Non-recurrent | 87 | 25.2 | 50.5 | 65.1 | 24.2 | 882.2 | 1047.2 | 17.3 |
| Average | 173 | 24.8 | 52.2 | 67.5 | 24.2 | 882.4 | 1051.2 | 17.4 |

Table B in S1 File. Details of mutation profile.

|  | C:G>T:A | C:G>G:C | C:G>A:T | A:T>T:A | A:T>C:G | A:T>G:C | 1bp ins | 2bp ins | 3bp ins | >3bp ins | 1bp del | 2bp del | 3bp del | >3bp del |
| --- | --- | --- | --- | --- | --- | --- | --- | --- | --- | --- | --- | --- | --- | --- |
| Recurrent (missense) | 395.60 | 121.77 | 87.34 | 42.21 | 69.59 | 166.14 |  |  |  |  |  |  |  |  |
| Non-recurrent (missense) | 391.05 | 123.54 | 89.07 | 42.55 | 70.06 | 165.95 |  |  |  |  |  |  |  |  |
| Total (missense) | 393.31 | 122.66 | 88.21 | 42.38 | 69.83 | 166.05 |  |  |  |  |  |  |  |  |
| Recurrent (frameshift) |  |  |  |  |  |  | 13.92 | 4.06 | 0.00 | 5.77 | 10.24 | 11.66 | 0.02 | 8.24 |
| Non-recurrent (frameshift) |  |  |  |  |  |  | 13.26 | 3.60 | 0.00 | 5.59 | 9.60 | 10.80 | 0.03 | 7.66 |
| Total (frameshift) |  |  |  |  |  |  | 13.59 | 3.83 | 0.00 | 5.68 | 9.92 | 11.23 | 0.03 | 7.95 |
| Recurrent (nonframeshift) |  |  |  |  |  |  | 0.41 | 0.00 | 6.79 | 8.99 | 0.03 | 0.01 | 39.92 | 13.84 |
| Non-recurrent (nonframeshift) |  |  |  |  |  |  | 0.43 | 0.00 | 6.15 | 9.21 | 0.02 | 0.00 | 37.16 | 12.13 |
| Total (nonframeshift) |  |  |  |  |  |  | 0.42 | 0.00 | 6.47 | 9.10 | 0.03 | 0.01 | 38.53 | 12.98 |
| Recurrent (nonsense) | 11.73 | 0.80 | 2.98 | 2.12 | 1.64 | 0.99 | 0.48 | 0.17 | 0.01 | 0.56 | 0.80 | 0.47 | 0.37 | 1.14 |
| Non-recurrent (nonsense) | 11.97 | 0.59 | 3.68 | 1.71 | 1.61 | 0.99 | 0.39 | 0.08 | 0.03 | 0.53 | 0.92 | 0.41 | 0.36 | 0.91 |
| Total (nonsense) | 11.85 | 0.69 | 3.33 | 1.91 | 1.62 | 0.99 | 0.43 | 0.13 | 0.02 | 0.54 | 0.86 | 0.44 | 0.36 | 1.02 |
| Recurrent (splicing) | 2.91 | 2.09 | 1.87 | 0.73 | 0.95 | 1.43 | 7.38 | 1.05 | 0.37 | 2.80 | 0.24 | 0.30 | 0.16 | 0.58 |
| Non-recurrent (splicing) | 2.89 | 2.20 | 1.74 | 0.63 | 0.97 | 1.63 | 8.13 | 1.20 | 0.48 | 2.34 | 0.32 | 0.24 | 0.25 | 0.67 |
| Total (splicing) | 2.90 | 2.14 | 1.80 | 0.68 | 0.96 | 1.53 | 7.76 | 1.12 | 0.43 | 2.57 | 0.28 | 0.27 | 0.21 | 0.62 |
| Recurrent (total) | 410.24 | 124.66 | 92.19 | 45.06 | 72.19 | 168.56 | 22.19 | 5.28 | 7.17 | 18.12 | 11.33 | 12.44 | 40.48 | 23.80 |
| Non-recurrent (total) | 405.90 | 126.32 | 94.48 | 44.90 | 72.63 | 168.57 | 22.21 | 4.87 | 6.67 | 17.67 | 10.86 | 11.46 | 37.80 | 21.36 |
| Total (total) | 408.06 | 125.50 | 93.34 | 44.98 | 72.41 | 168.57 | 22.20 | 5.08 | 6.92 | 17.89 | 11.09 | 11.95 | 39.13 | 22.57 |

Table C in S1 File. Summary of mutation profile in hypermutated samples.

| **Sample Group** | **# of samples** | **Splicing** | **Frameshift indel** | **Nonframeshift indel** | **Nonsense** | **Missense** | **Total** | **Mutations/Mb** |
| --- | --- | --- | --- | --- | --- | --- | --- | --- |
| Overall | 13 | 1476 | 7656 | 3545 | 3637 | 77403 | 93717 | 1548.8 |
| Recurrent | 5 | 57.2 | 720.4 | 171.6 | 124.6 | 1873.0 | 2946.8 | 48.7 |
| Non-recurrent | 8 | 51.5 | 360.0 | 87.8 | 153.6 | 2107.5 | 2760.4 | 45.6 |
| Average | 13 | 53.7 | 498.6 | 120.0 | 142.5 | 2017.3 | 2832.1 | 47.2 |

Table D in S1 File. Details of mutation profile in hypermutated samples.

|  | C:G>T:A | C:G>G:C | C:G>A:T | A:T>T:A | A:T>C:G | A:T>G:C | 1bp ins | 2bp ins | 3bp ins | >3bp ins | 1bp del | 2bp del | 3bp del | >3bp del |
| --- | --- | --- | --- | --- | --- | --- | --- | --- | --- | --- | --- | --- | --- | --- |
| Recurrent (missense) | 804.40 | 243.20 | 205.20 | 175.20 | 165.40 | 308.80 |  |  |  |  |  |  |  |  |
| Non-recurrent (missense) | 1017.63 | 141.75 | 424.00 | 56.00 | 175.50 | 311.75 |  |  |  |  |  |  |  |  |
| Total (missense) | 935.62 | 180.77 | 339.85 | 101.85 | 171.62 | 310.62 |  |  |  |  |  |  |  |  |
| Recurrent (frameshift) |  |  |  |  |  |  | 139.40 | 54.40 | 0.00 | 93.20 | 277.40 | 69.80 | 1.20 | 76.40 |
| Non-recurrent (frameshift) |  |  |  |  |  |  | 69.75 | 7.50 | 0.00 | 4.38 | 236.50 | 25.75 | 0.25 | 10.00 |
| Total (frameshift) |  |  |  |  |  |  | 96.54 | 25.54 | 0.00 | 38.54 | 252.23 | 42.69 | 0.62 | 35.54 |
| Recurrent (nonframeshift) |  |  |  |  |  |  | 0.80 | 0.00 | 29.00 | 30.60 | 0.40 | 0.20 | 77.80 | 35.20 |
| Non-recurrent (nonframeshift) |  |  |  |  |  |  | 0.50 | 0.00 | 8.50 | 10.13 | 0.75 | 0.13 | 53.50 | 16.63 |
| Total (nonframeshift) |  |  |  |  |  |  | 0.62 | 0.00 | 16.38 | 18.00 | 0.62 | 0.15 | 62.85 | 23.77 |
| Recurrent (nonsense) | 36.20 | 4.20 | 17.60 | 16.60 | 3.00 | 3.20 | 5.60 | 2.40 | 2.00 | 13.20 | 11.80 | 4.00 | 1.20 | 5.60 |
| Non-recurrent (nonsense) | 59.38 | 1.00 | 70.63 | 2.50 | 5.13 | 2.63 | 2.00 | 0.00 | 0.00 | 0.75 | 8.63 | 1.13 | 0.88 | 1.38 |
| Total (nonsense) | 50.46 | 2.23 | 50.23 | 7.92 | 4.31 | 2.85 | 3.38 | 0.92 | 0.77 | 5.54 | 9.85 | 2.23 | 1.00 | 3.00 |
| Recurrent (splicing) | 9.40 | 6.00 | 6.80 | 1.60 | 2.00 | 10.40 | 8.00 | 1.00 | 0.80 | 4.80 | 2.00 | 1.00 | 0.60 | 1.80 |
| Non-recurrent (splicing) | 10.63 | 2.63 | 13.50 | 0.25 | 1.75 | 9.00 | 6.63 | 0.75 | 0.63 | 2.38 | 0.88 | 0.88 | 0.38 | 1.00 |
| Total (splicing) | 10.15 | 3.92 | 10.92 | 0.77 | 1.85 | 9.54 | 7.15 | 0.85 | 0.69 | 3.31 | 1.31 | 0.92 | 0.46 | 1.31 |
| Recurrent (total) | 861.00 | 254.00 | 231.00 | 195.60 | 170.00 | 324.20 | 156.40 | 57.80 | 31.80 | 142.40 | 296.00 | 76.00 | 80.80 | 120.40 |
| Non-recurrent (total) | 1101.13 | 147.00 | 515.00 | 59.38 | 182.88 | 326.38 | 80.88 | 8.38 | 9.13 | 17.88 | 251.50 | 27.88 | 55.00 | 29.38 |
| Total (total) | 1008.77 | 188.15 | 405.77 | 111.77 | 177.92 | 325.54 | 109.92 | 27.38 | 17.85 | 65.77 | 268.62 | 46.38 | 64.92 | 64.38 |


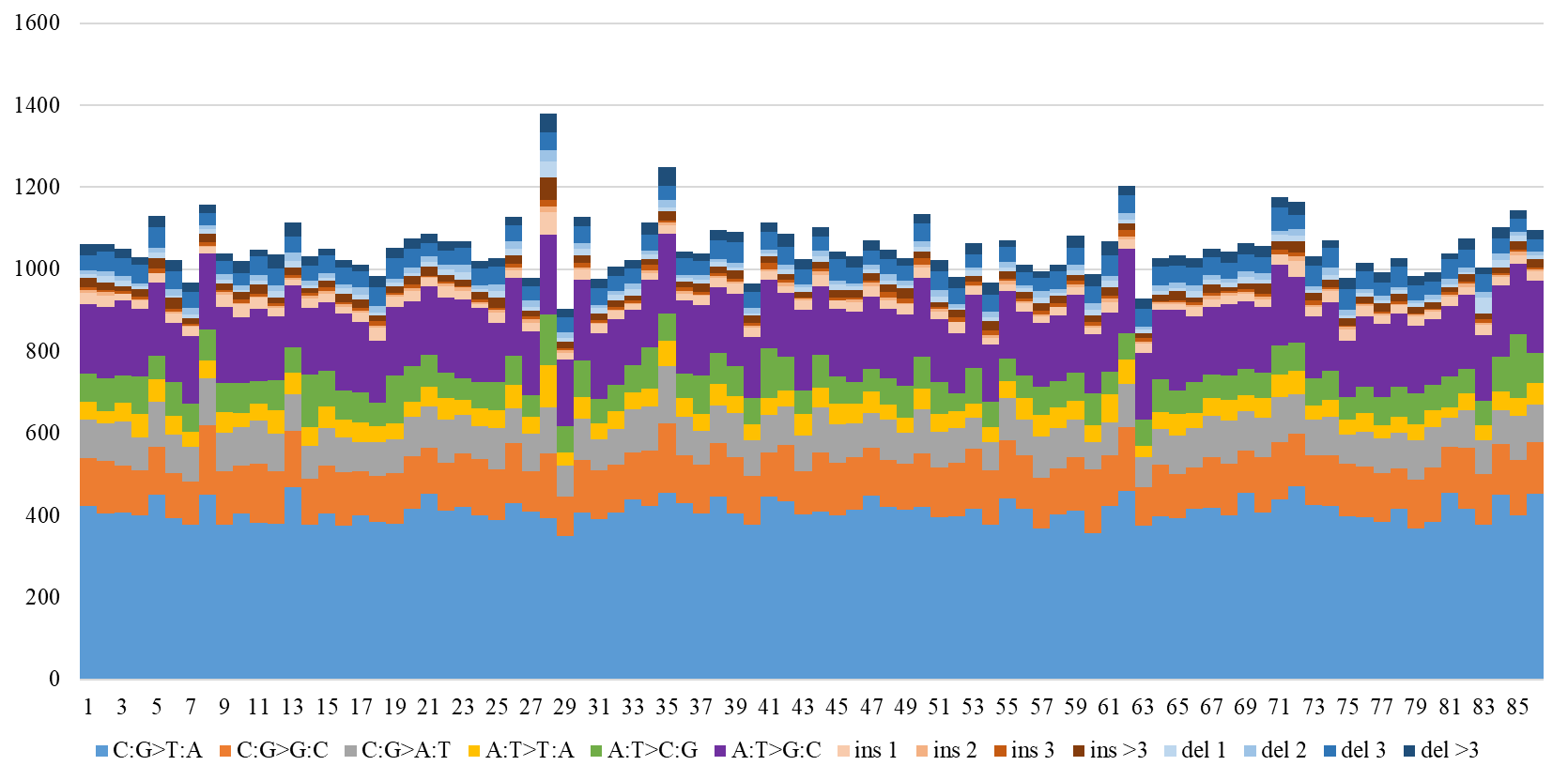


Figure A in S1 File. Mutation spectrum of samples in recurrence group.


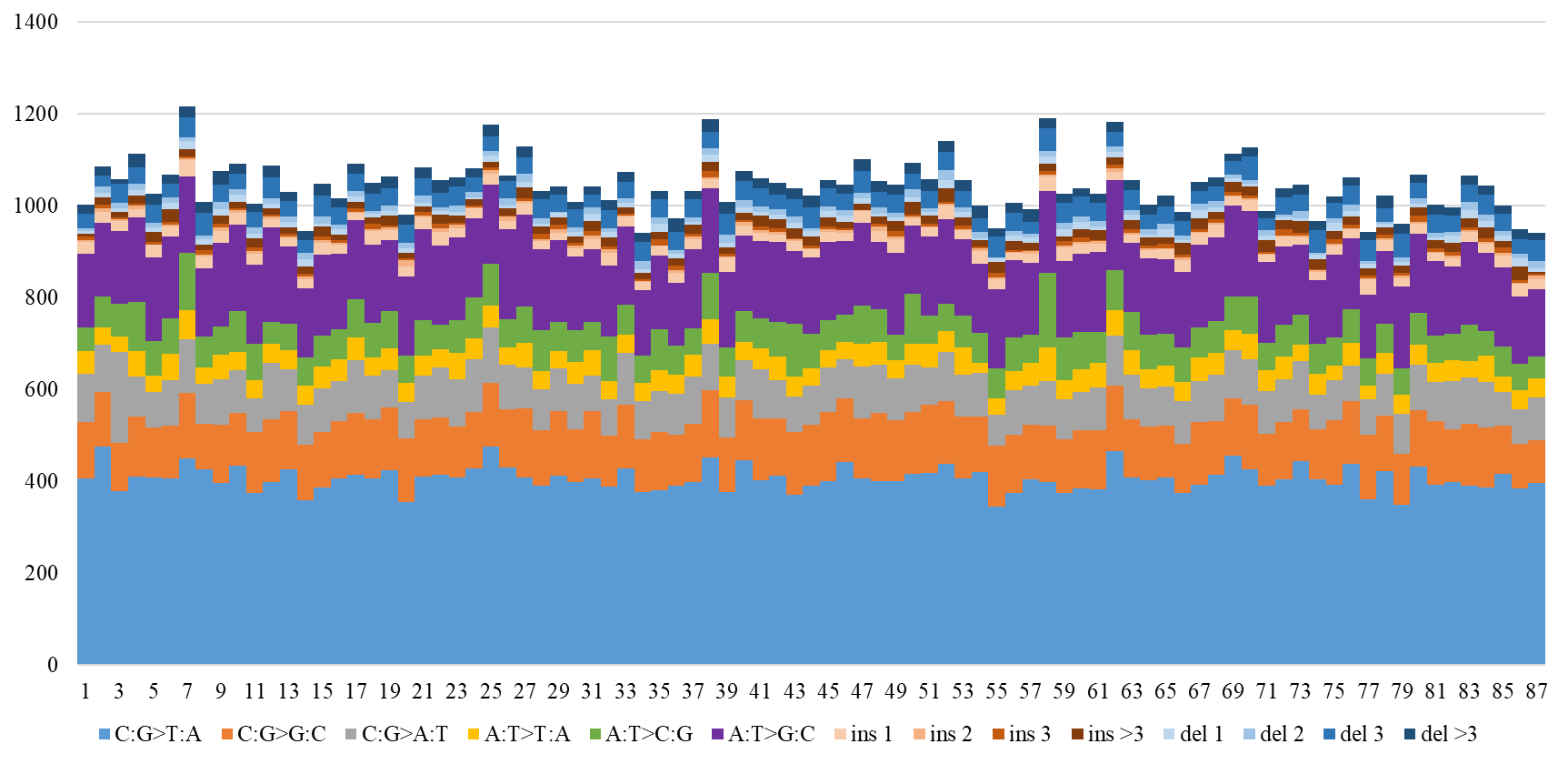


Figure B in S1 File. Mutation spectrum of samples in non-recurrence group.


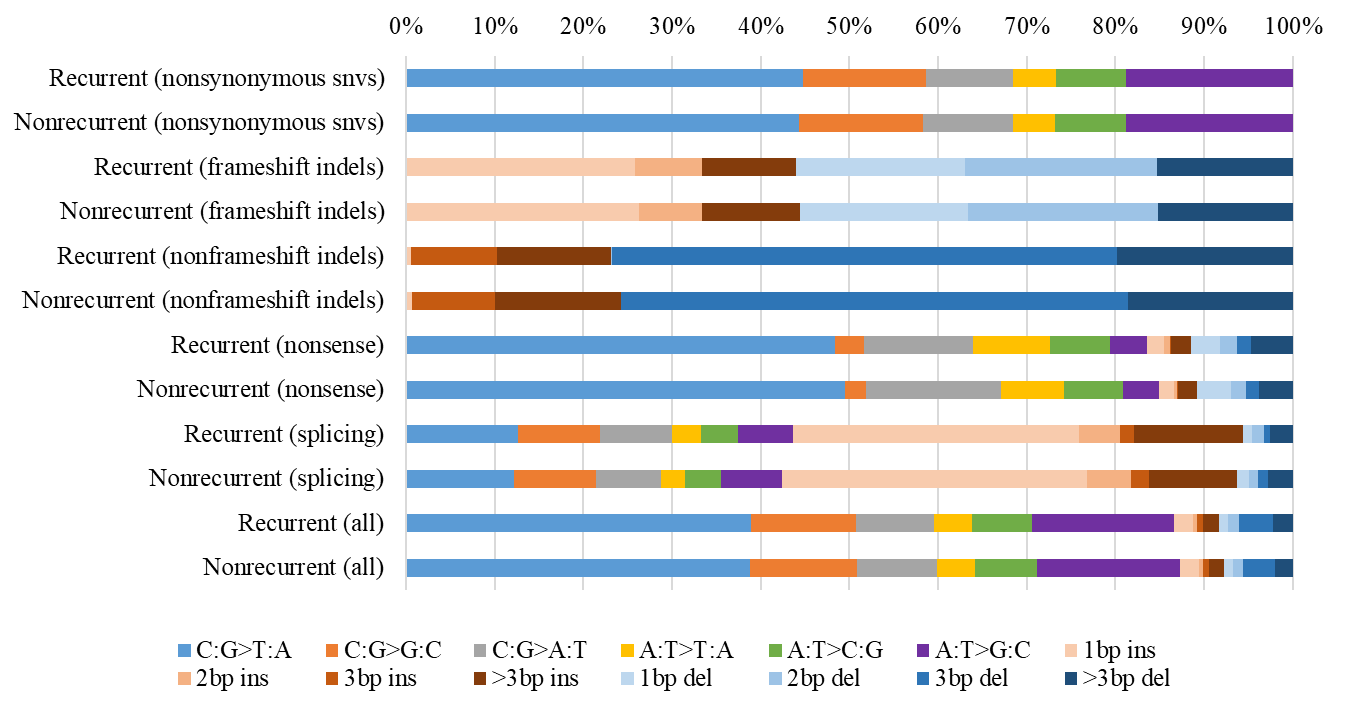


Figure C in S1 File. Mutation spectrum of samples.


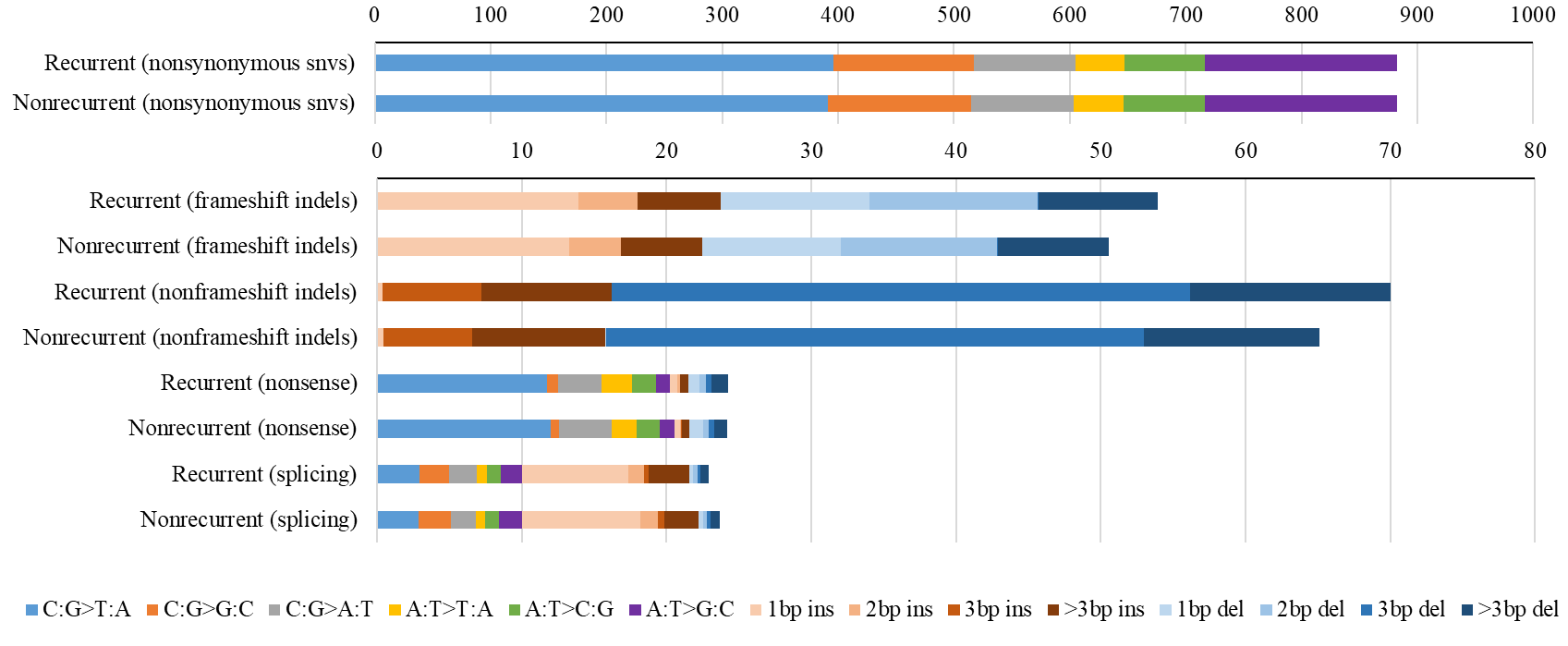


Figure D in S1 File. Average count of mutations per sample by mutation type.


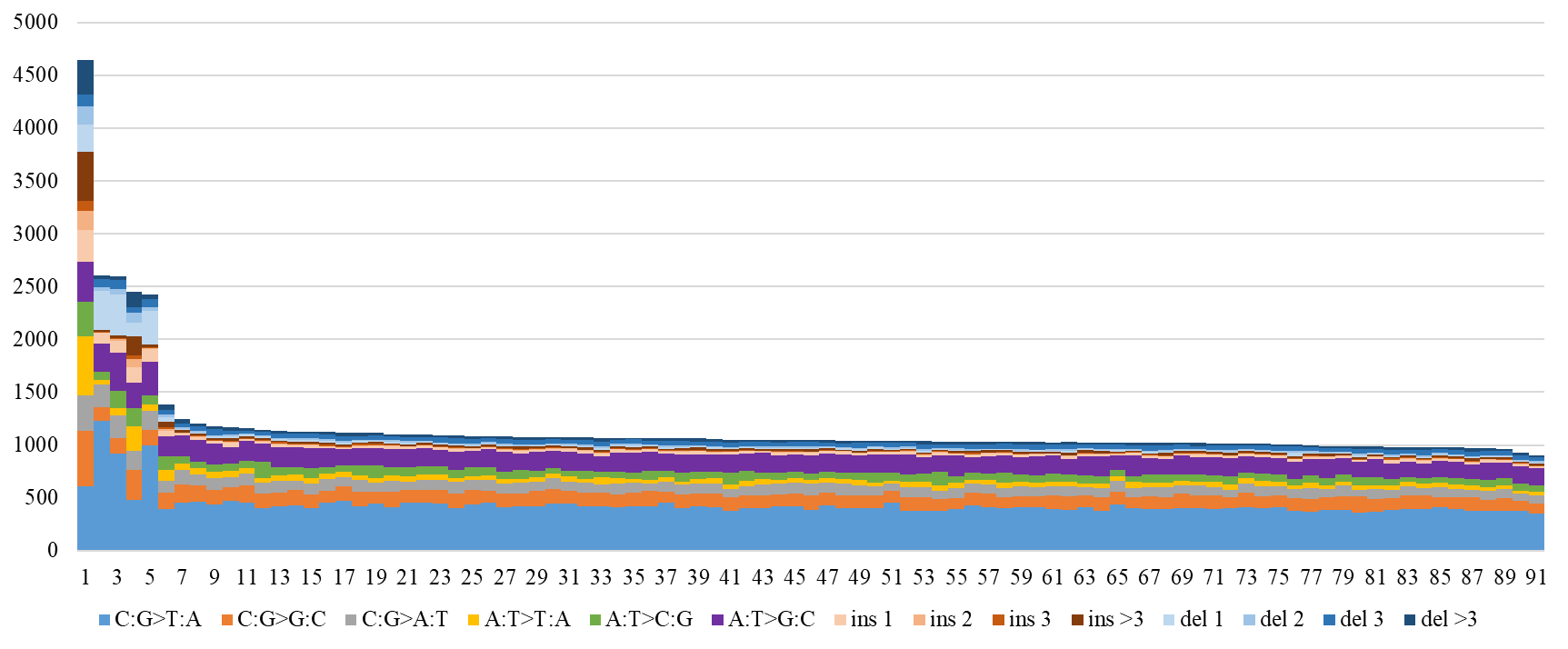


Figure E in S1 File. Mutation spectrum of samples in recurrence group sorted by TMB.


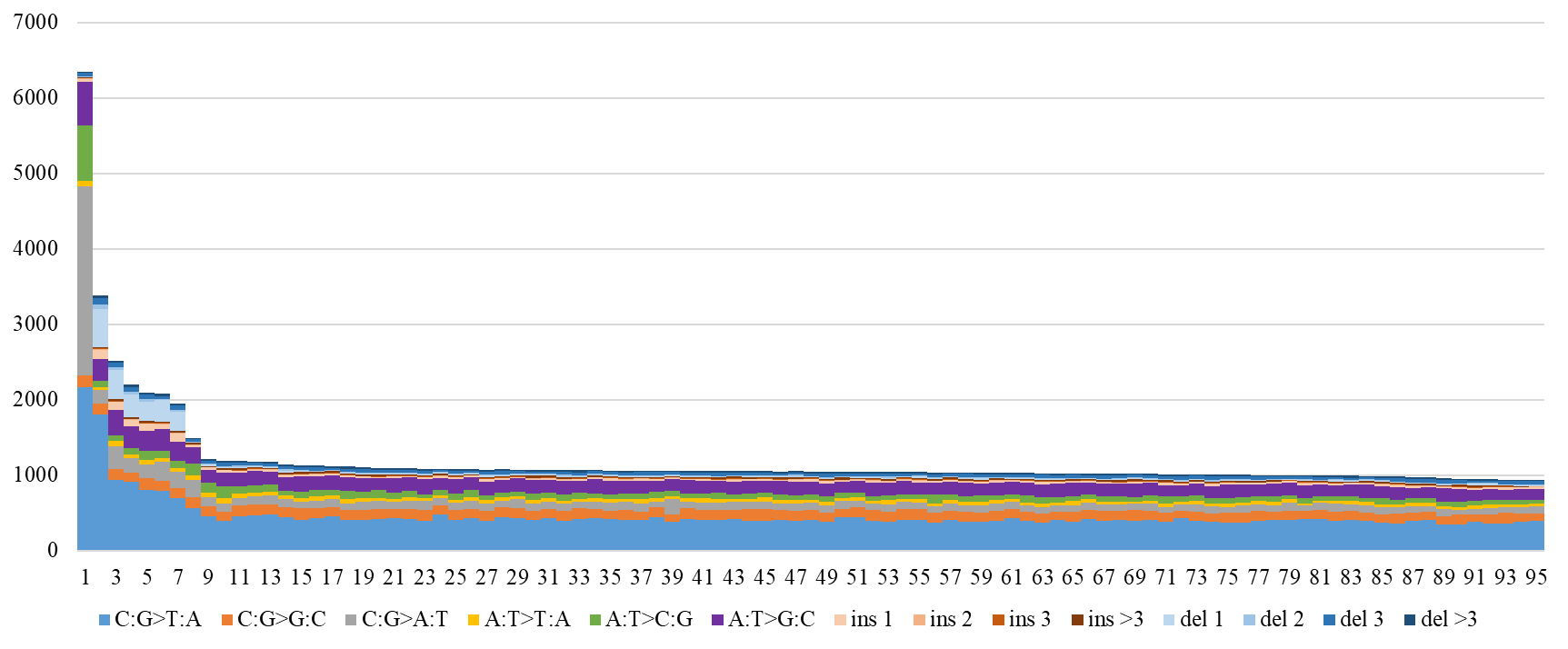


Figure F in S1 File. Mutation spectrum of samples in non-recurrence group sorted by TMB.


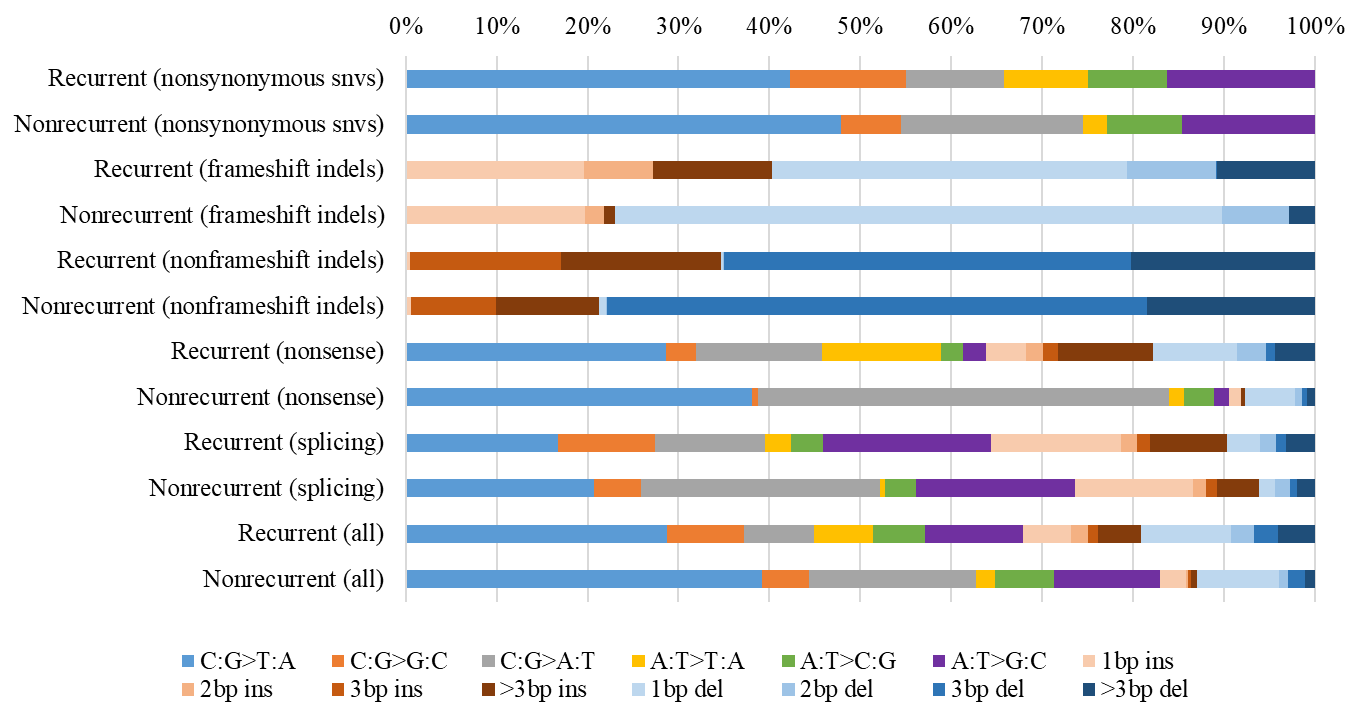


Figure G in S1 File. Mutation spectrum of hypermutated samples.


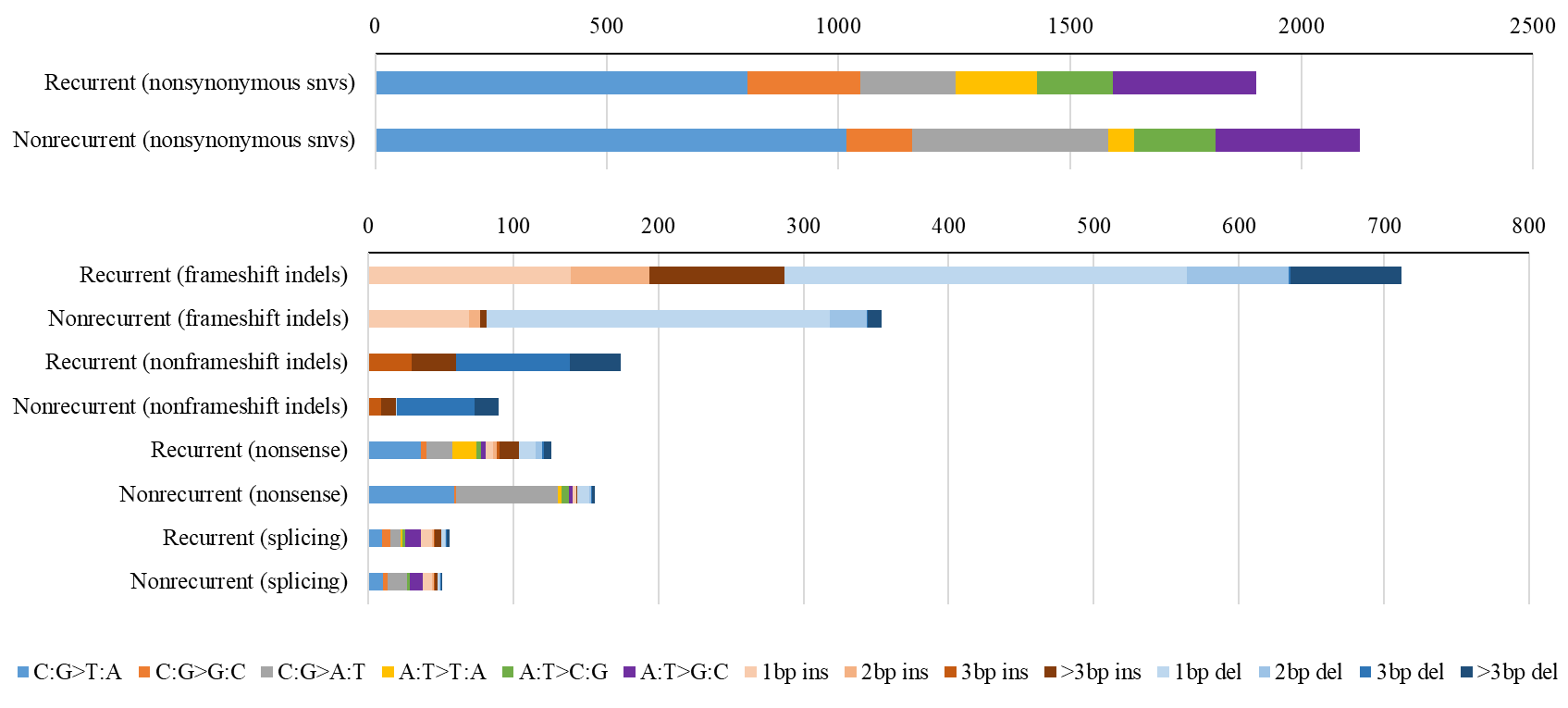


Figure H in S1 File. Average count of mutations per sample by mutation type in hypermutated samples.
