# Supplementary material for: Genetic variations and recurrence in stage III Korean colorectal cancer: Insights from tumor-only mutation analysis
Source: PLoS One. 2025 May 23;20(5):e0323302. doi: 10.1371/journal.pone.0323302 (PMC12101642; doi:10.1371/journal.pone.0323302)
Supplement: S2 File — (DOCX) [file pone.0323302.s009.docx]

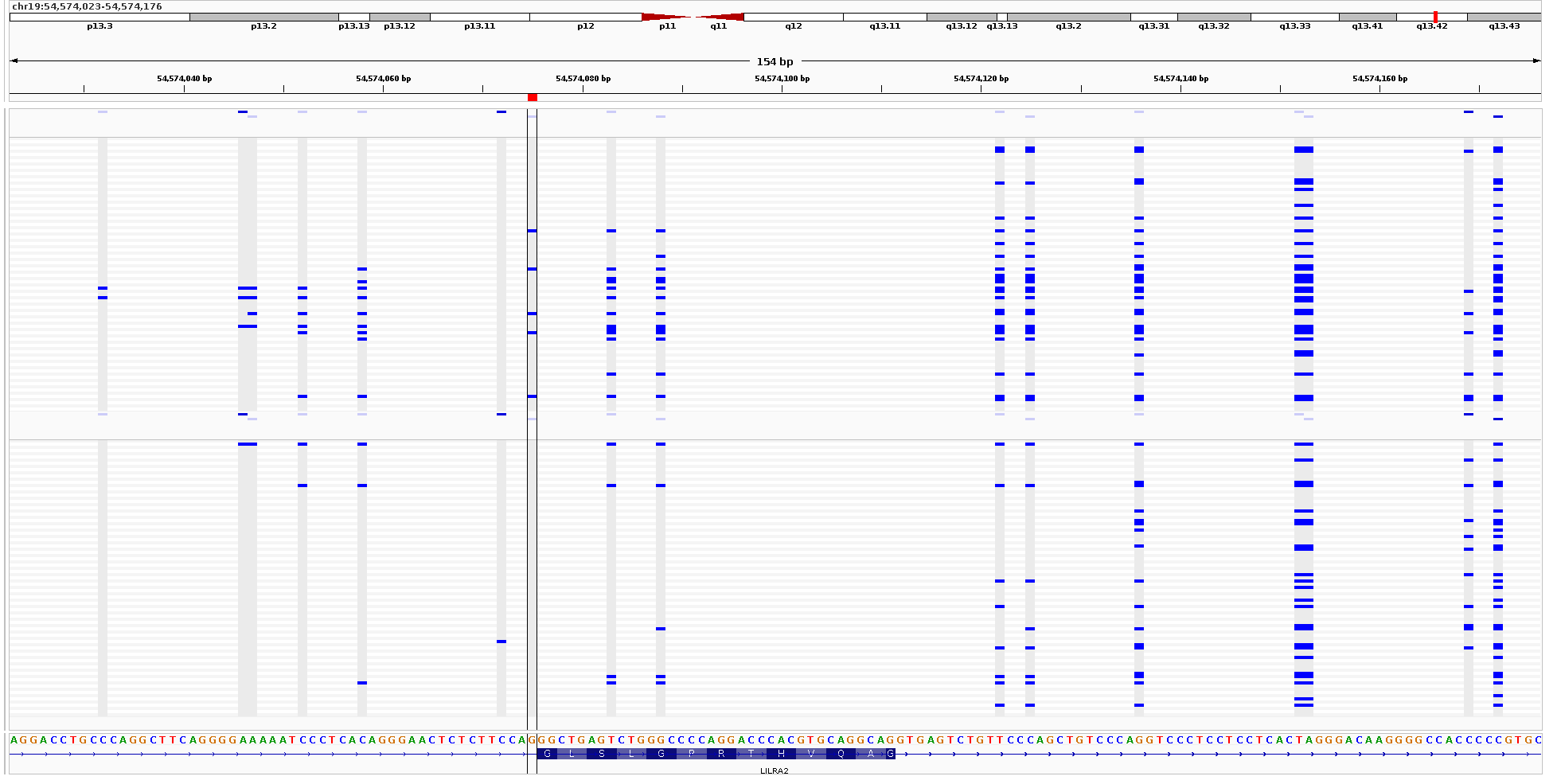


Figure A in S2 File. Statistically significant mutation in splice site in LILRA2 gene. The horizontal line in the center divides the figure with the recurrent group above and the nonrecurrent group below. The blue short line denotes the variant in the position. The mutation position (chr19:54574075) is highlighted with vertical lines. As shown in the figure, 5 samples in recurrent group showed the mutation but none in nonrecurrent group showed the mutation.


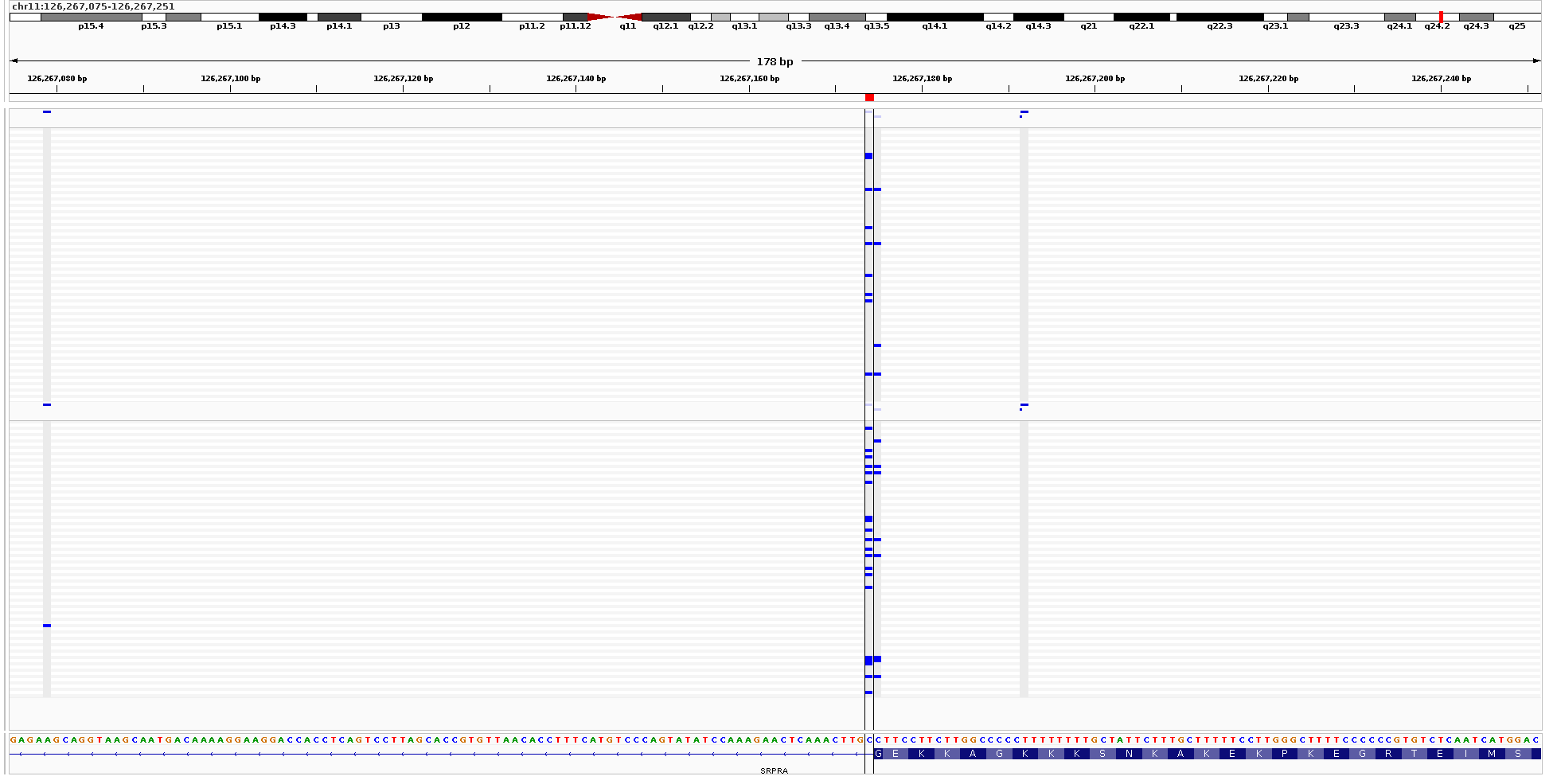


Figure B in S2 File. Statistically significant mutation in the splice site in SRPRA gene. The horizontal line in the center divides the figure with the recurrent group above and the nonrecurrent group below. The blue short line denotes the variant in the position. The mutation position (chr11:126267174) is highlighted with vertical lines. As shown in the figure, 9 samples in recurrent group showed the mutation and 20 samples in nonrecurrent group showed the mutation.


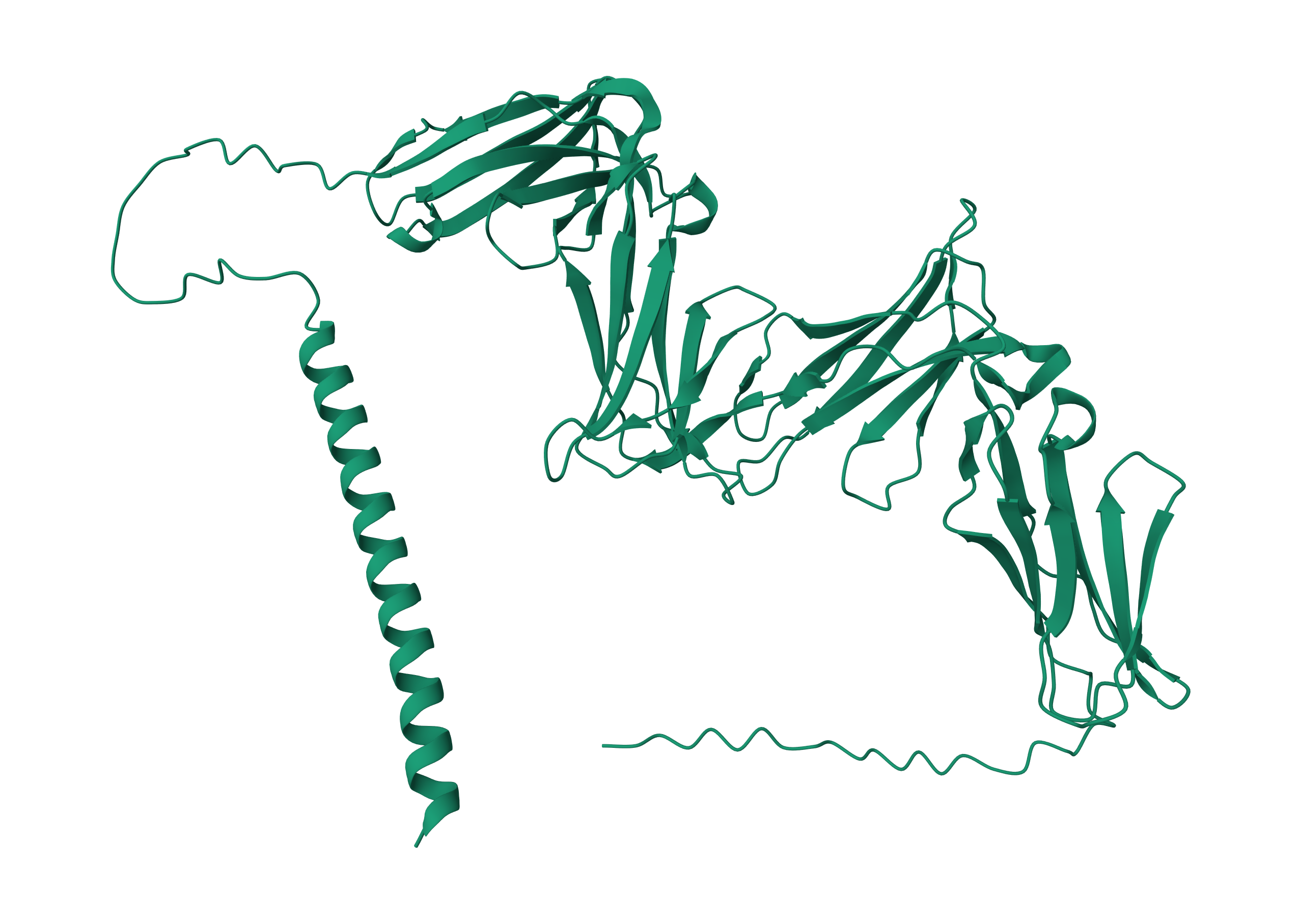


Figure C in S2 File. Protein structure of the LILRA2 gene.


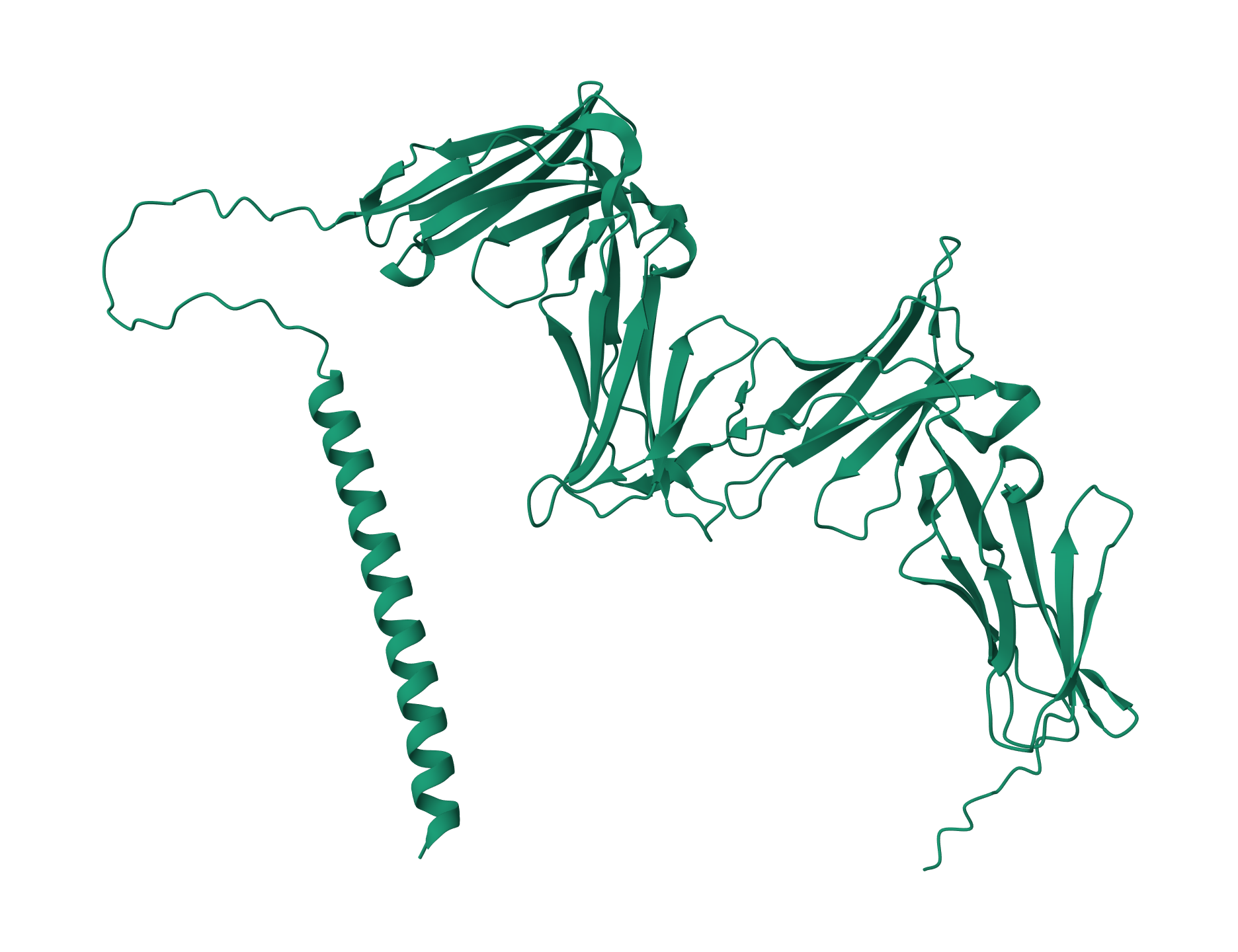


Figure D in S2 File. Protein structure of the LILRA2 gene with exon skipping.


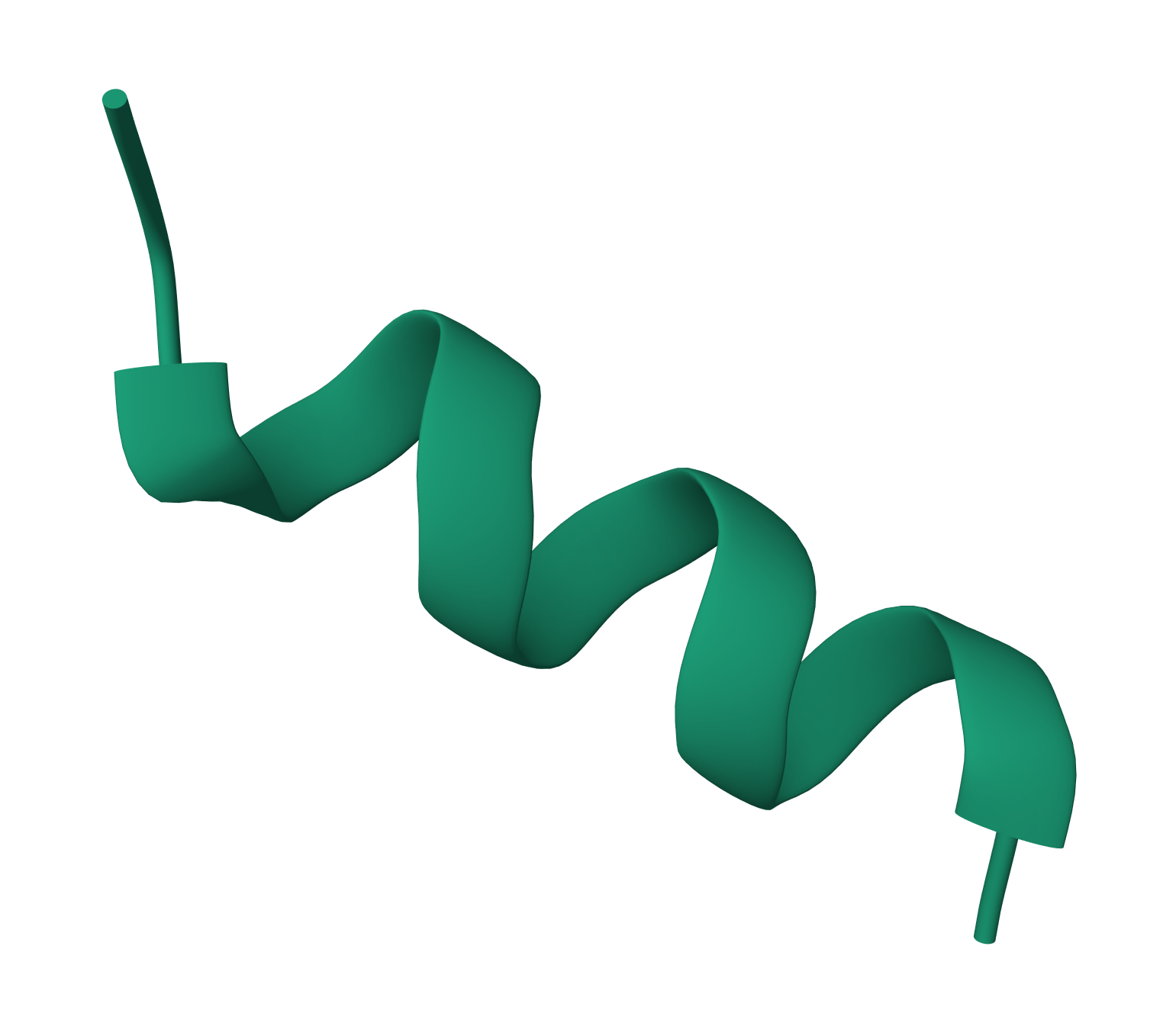


Figure E in S2 File. Protein structure of the LILRA2 gene with intron retention.


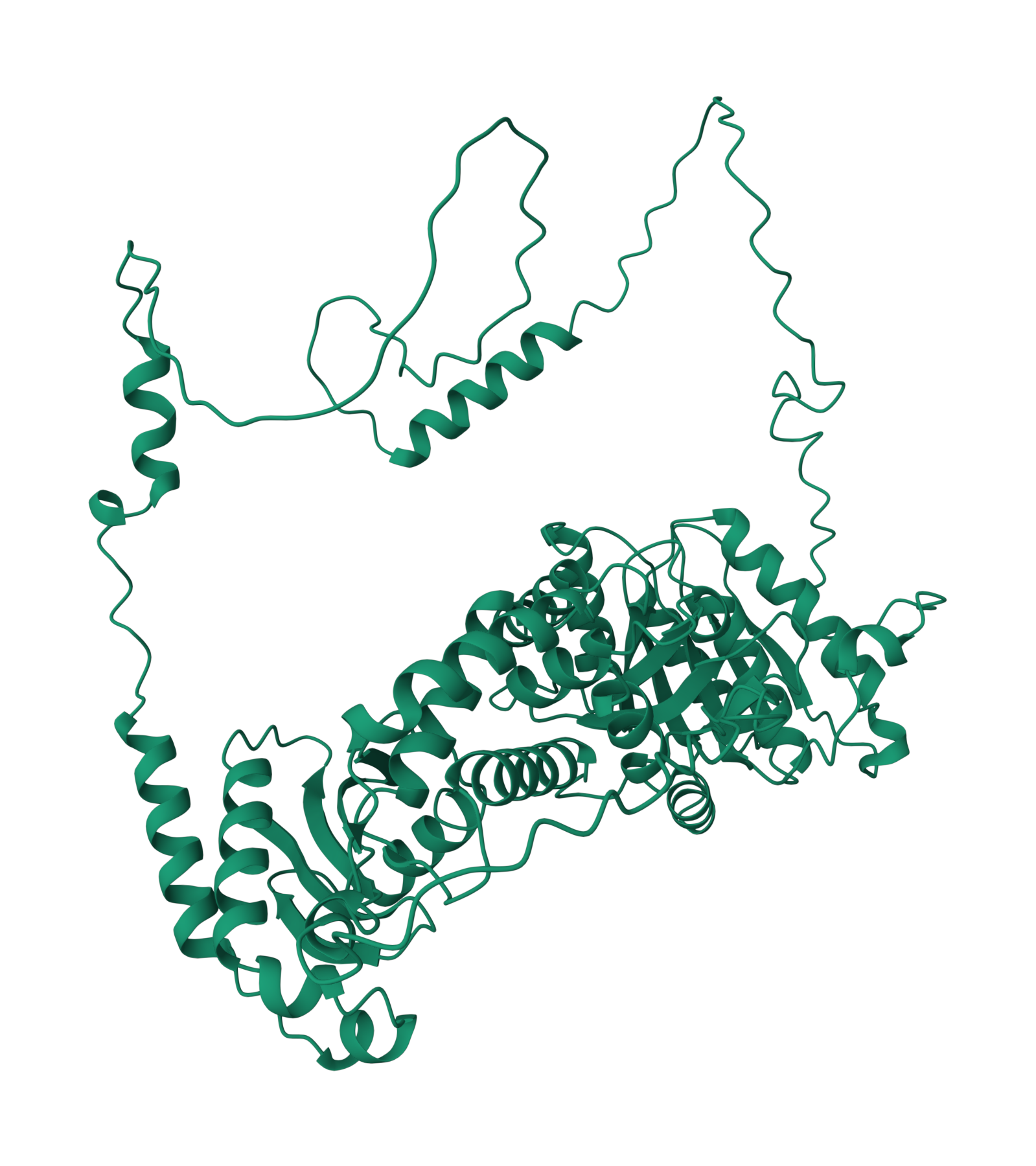


Figure F in S2 File. Protein structure of the SRPRA gene.


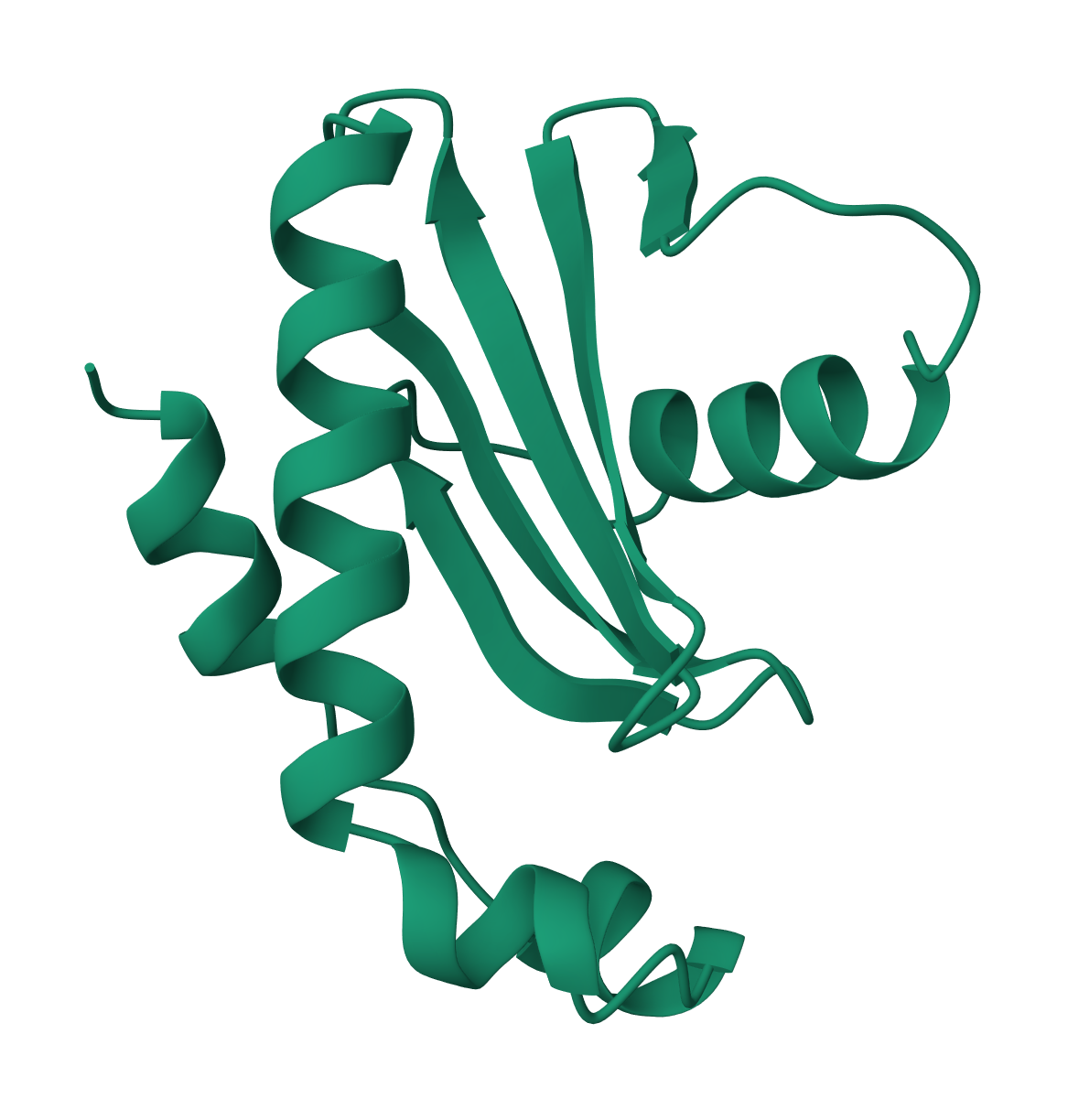


Figure G in S2 File. Protein structure of the SRPRA gene with exon skipping.


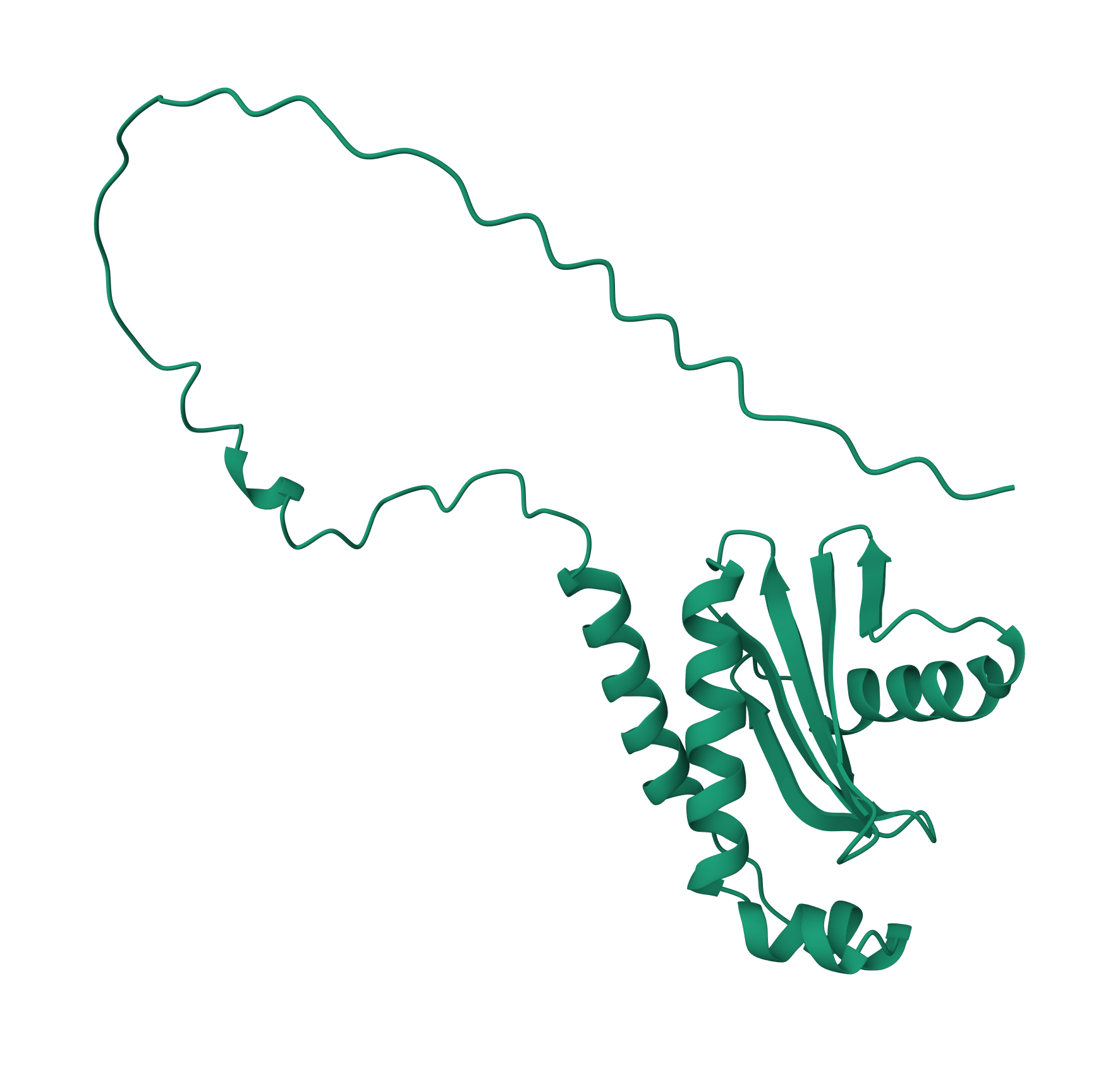


Figure H in S2 File. Protein structure of the SRPRA gene with intron retention.
