# Supplementary material for: Genetic variations and recurrence in stage III Korean colorectal cancer: Insights from tumor-only mutation analysis
Source: PLoS One. 2025 May 23;20(5):e0323302. doi: 10.1371/journal.pone.0323302 (PMC12101642; doi:10.1371/journal.pone.0323302)
Supplement: S3 File — (DOCX) [file pone.0323302.s010.docx]

Table A in S3 File. Target molecules of each network.

| **No.** | **Molecules** |
| --- | --- |
| 1 | Akt, BPTF, CBX2, CFH, CGB3 (includes others), CNTNAP4, Cofilin, DSG2, ERK1/2, FCGR1A, GNAQ, HLA-A, HLA-B, HLA-C, HLA-DRB1, IgG, Immunoglobulin, ITGAV, ITGB5, KIR, KRT10, LETM1, MAP3K10, MAPKAPK2, MUC4, MUC5AC, P38 MAPK, PPP1R15A, SCRIB, SIGMAR1, SRC (family), STK4, SYNE2, TAPBPL, TRIM15 |
| 2 | ADGRB1, ARHGAP28, ARHGEF5, BEX3, CFTR, CG, E2F7, ERK, ESRRA, FOXF1, FYB1, GOLGA6A (includes others), Histone h3, Hsp90 (family), Jnk, KDM6B, KMT2C, Mek, MYH14, MYL1, NANOG, NBPF10 (includes others), NCL, NFkB (complex), PHOX2B, PKN3, PRSS2, RNA polymerase II, RXRB, TCR, TICAM1, TTN, UACA, ZFP64, ZNF83 |
| 3 | APOA1, ARID1A, BCAS1, BCL2, C4A/C4B, CASP14, CKB, DHX16, EML4-ALK, EPPK1, FLG, FRZB, HPX, KCNN3, LGALS7/LGALS7B, LSR, MOGS, MTARC1, NCOA2, NCOR2, NME1, OCIAD2, POF1B, PROM1, PRSS3, RGMA, RSU1, SERPINB6, SNCA, SRPRA, SRRT, THRB, USP17L2 (includes others), ZNF185, ZNF774 |
| 4 | ANK3, APLF, ATM, BRCC3, CARD8, CCNK, CDK12, CEP170B, CHD2, CUBN, DDX19B, FANCD2, FANCI, FBXO3, GPATCH3, GSTA2, MDC1, MEFV, MMS22L, NLRP3, NUPR1, PARP1, PCTP, PIM1, POLA2, POLH, RCAN3, RFC5, RNF4, TEP1, TIMELESS, UNC5B, USP37, XRCC1, ZDHHC13 |
| 5 | AHNAK2, CABIN1, CCND1, CTBS, DPF3, EFNA3, ENDOD1, FOXJ2, FUS-DDIT3, G3BP1, GAS2L3, GLI1, GPC6, HIF1A, HRNR, IL6, IRF2BPL, KLF13, LAMB2, LIFR, LILRA2, mir-338, MTMR1, NCAPD3, NQO2, PAH, PGR, PRRC2A, R3HCC1L, RNF170, RPL27A, SPRR3, SS18L1, TMEM79, TPSD1 |
| 6 | ALPK1, AMER2, ATP6AP2, CCDC88C, CTNNB1, DAAM1, DYNLRB1, FEZ2, FOXQ1, GALNT6, GPS1, MIXL1, MRC2, MUC13, MUC5B, MUC6, PKD1, PORCN, PRICKLE1, QPCT, RHOU, RNF39, RNMT, RPRD1A, SATB1, SCG5, SERPINA3, SLC27A2, STAT5A, TDG, TGFB1, TLE4, TUBA4A, WBP2, WLS |
| 7 | ADA, ADA2, ANKS1B, BCL2L2, CD3, CD40LG, CD6, CHD3, CXCR4, EDN2, GRHL3, H6PD, HIRA, HLA-DQA1, HLA-DQA2, HLA-DQB1, HLA-DRB5, IFNG, KLF4, KRT78, LAD1, LILRA1, MRPS18B, NDFIP2, NR3C1, PAX3, PIM3, PLAGL1, RIGI, RNF38, SPTB, TNFAIP8, TNS4, TP63, TTC28 |
| 8 | BMP2K, CACNA1A, CTNNB1, DPEP1, DUOXA1, EPAS1, FUT3, GFM2, HACL1, HNF1A, HOMER3-AS1, HSD17B2, JAM3, KCTD9, MIR3662, MYC, NAGS, NTRK1, PDE10A, PHGDH, RNF32-DT, RPUSD1, SDCBP2-AS1, SLC25A5, SNRK, SOX15, SPOCK2, SUMO1P3, TGFB2, TLE2, USH1C, USP17L22 (includes others), USP33, ZNF300, ZNF469 |
| 9 | AGO1, ATXN7L1, BARX2, CDKN3, DMXL1, DSG1, DYNLL1, EIF4G2, EIF5A2, ERBB4, ESR1, GJD3, HNRNPD, HSP90B1, IFI16, INHBA, KRTAP10-6, MAST2, mir-19, MPEG1, NME1, NOTCH3, PIN1, SF3A2, SF3B2, SFPQ, SLC2A4, TAGLN, TH, TOP1, VAV3, ZBTB10, ZNF24, ZNF479, ZNF500 |


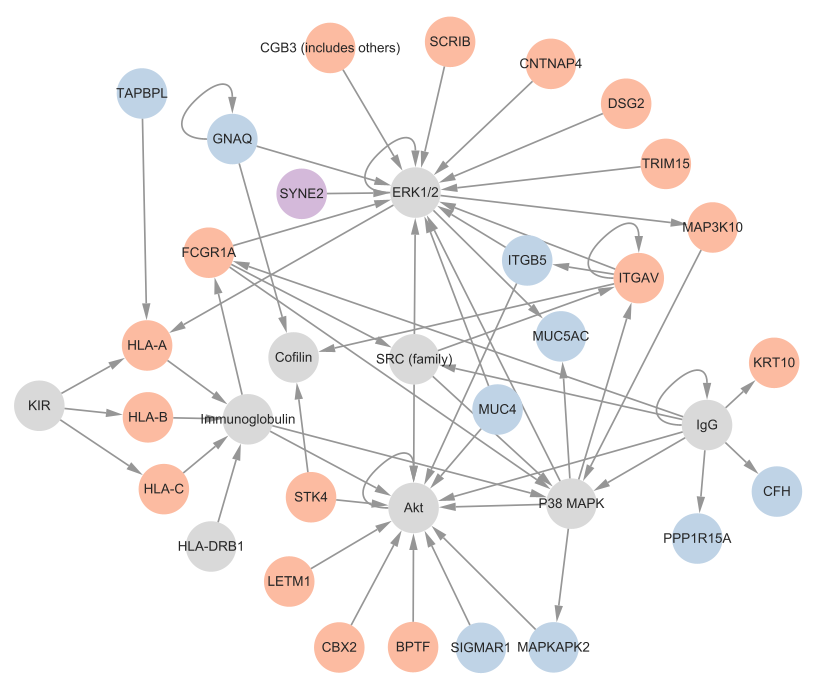


Figure A in S3 File. Protein-protein interaction of network 1.


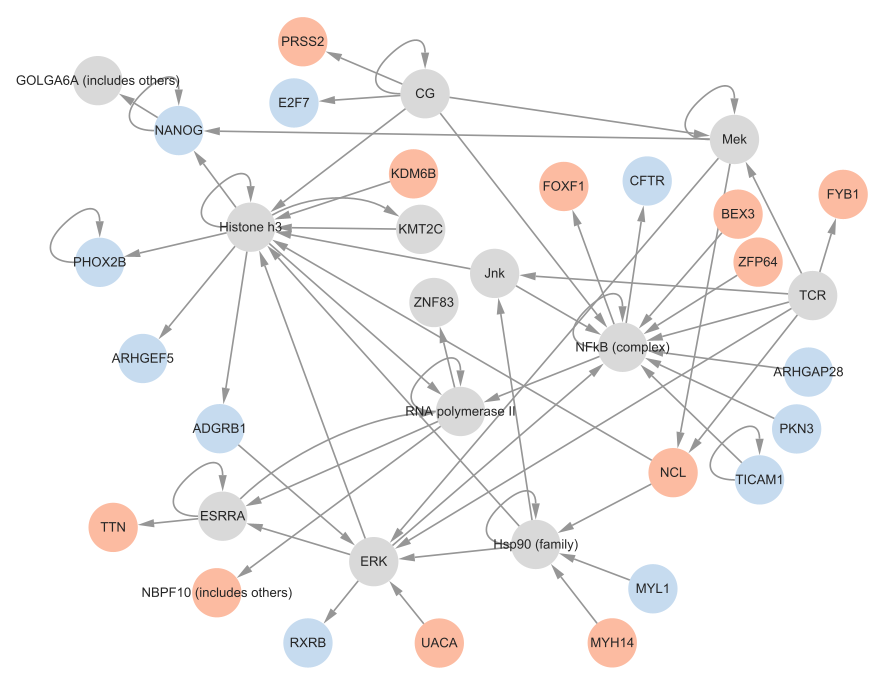


Figure B in S3 File. Protein-protein interaction of network 2.


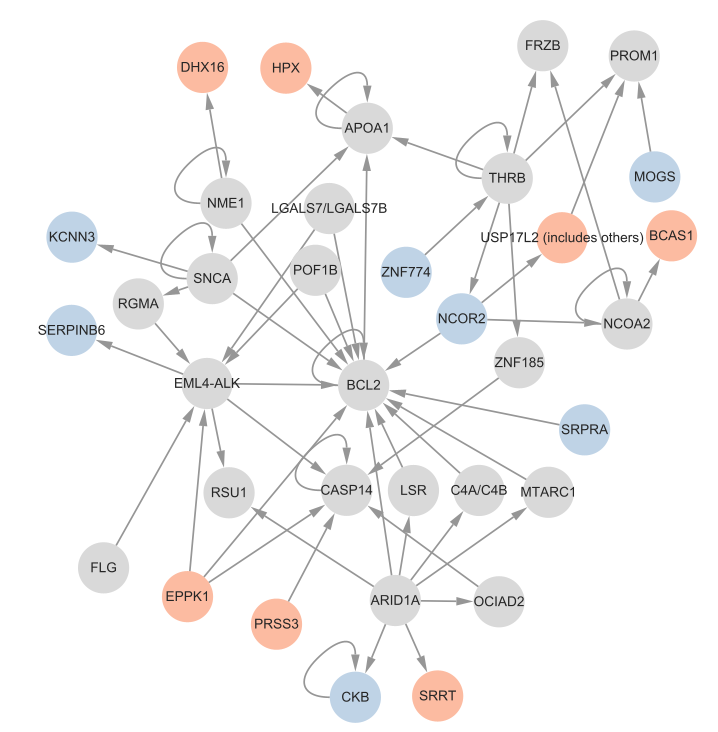


Figure C in S3 File. Protein-protein interaction of network 3.


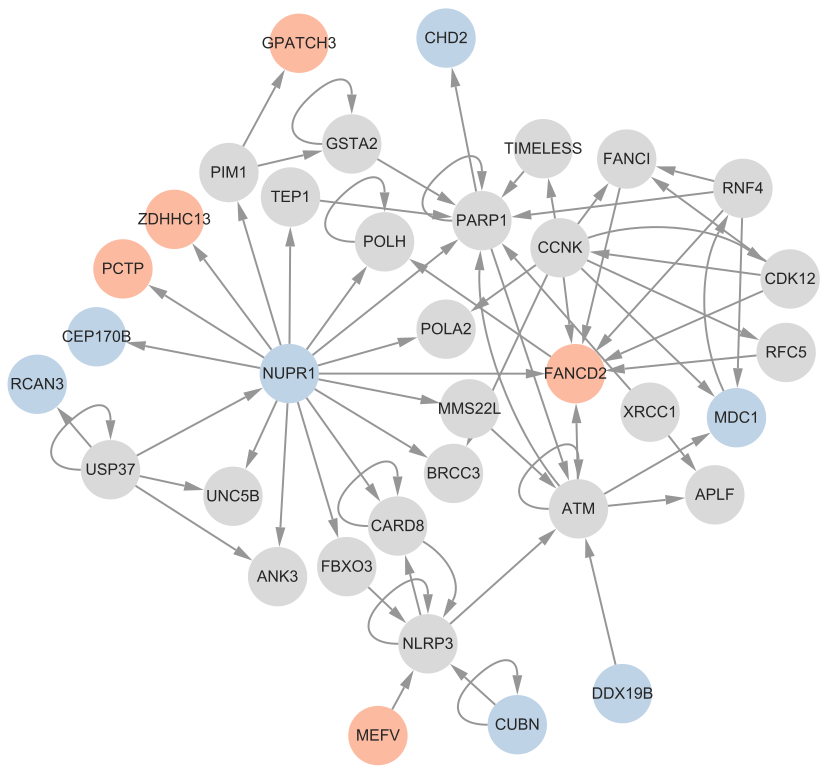


Figure D in S3 File. Protein-protein interaction of network 4.


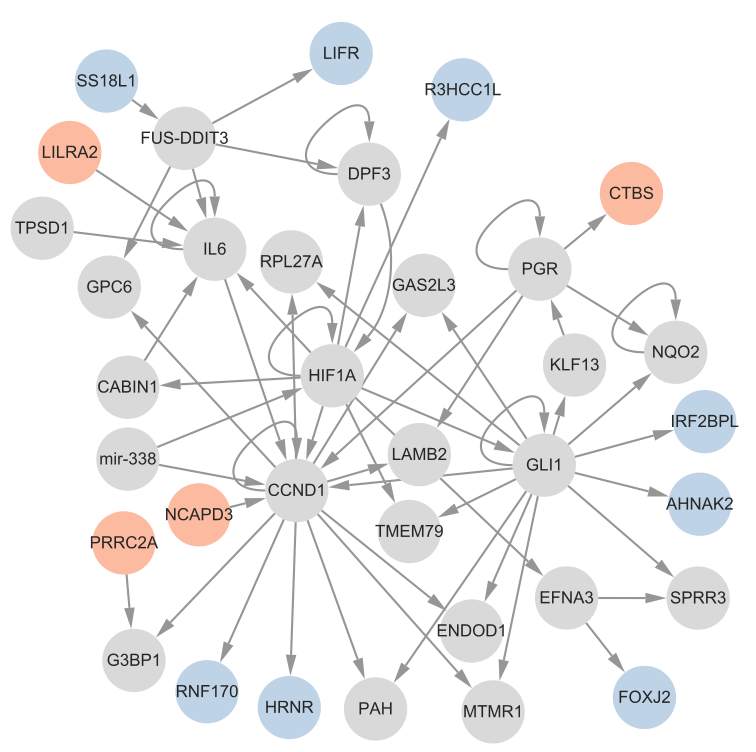


Figure E in S3 File. Protein-protein interaction of network 5.


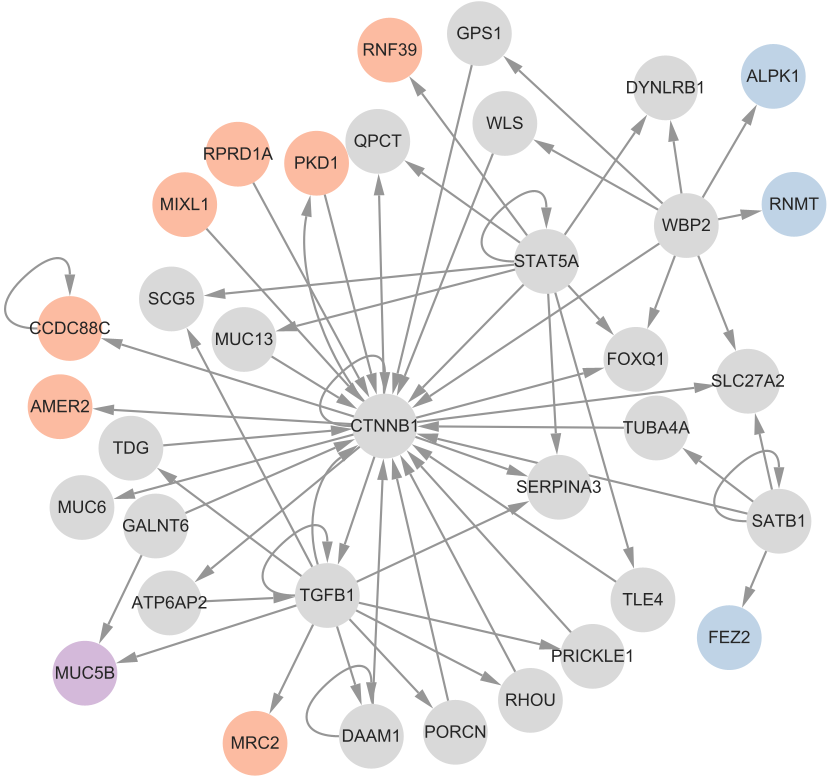


Figure F in S3 File. Protein-protein interaction of network 6.


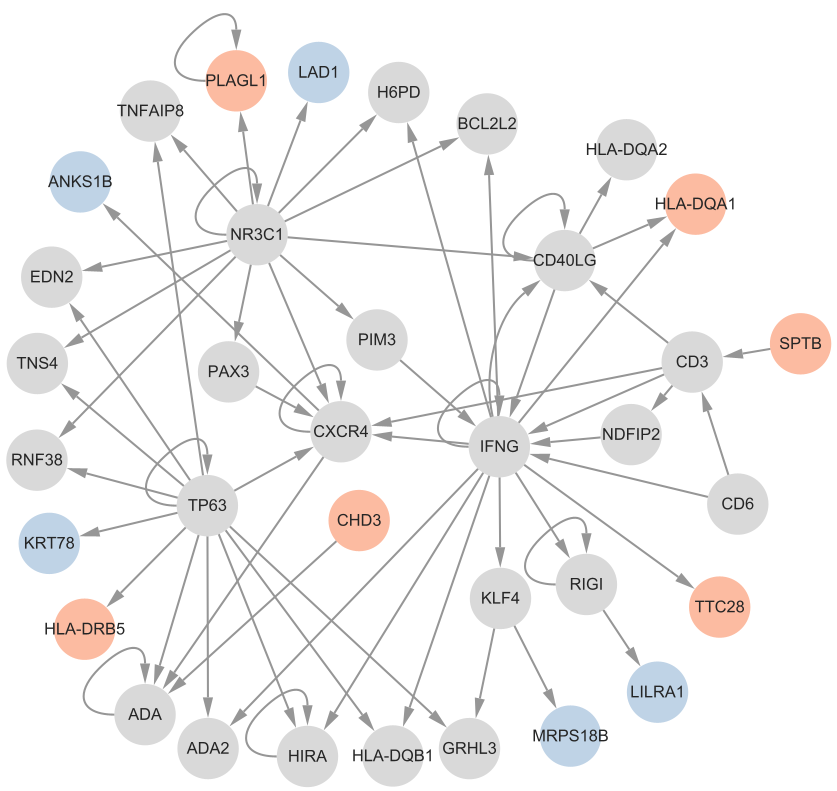


Figure G in S3 File. Protein-protein interaction of network 7.


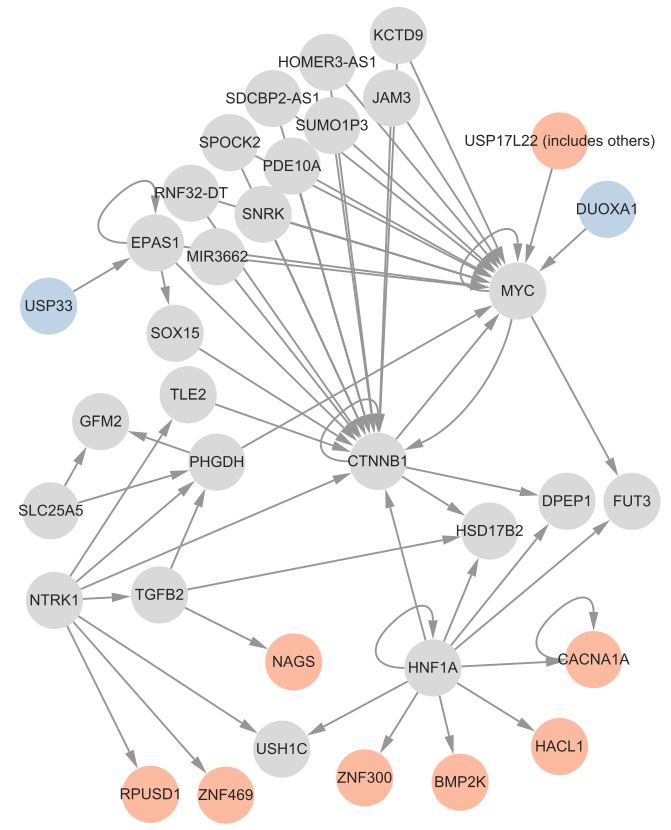


Figure H in S3 File. Protein-protein interaction of network 8.


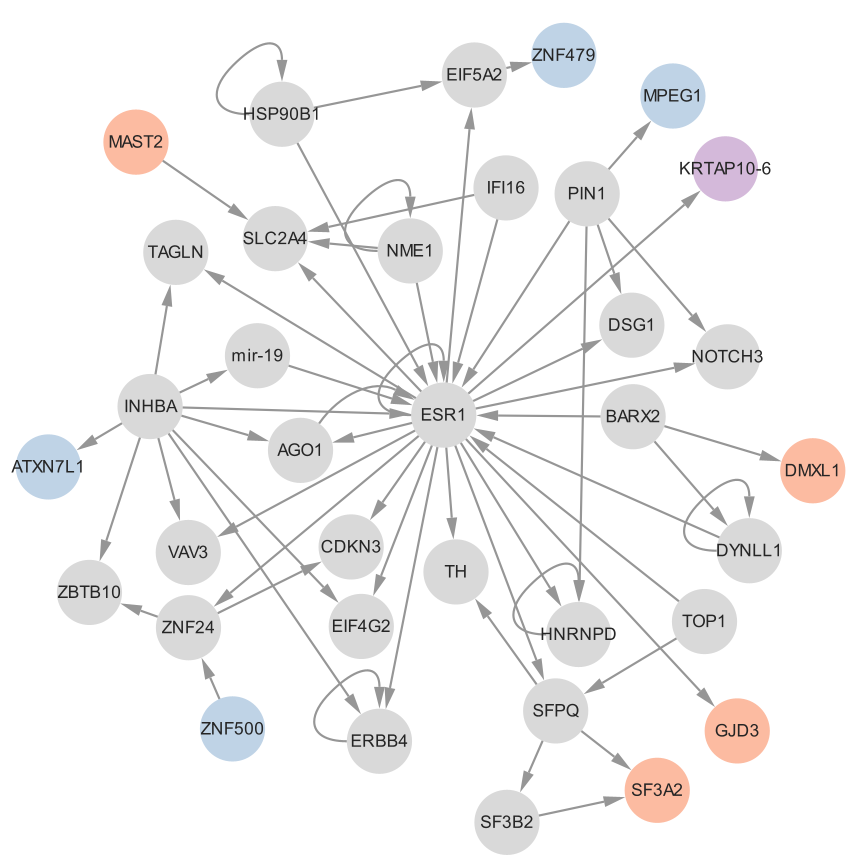


Figure I in S3 File. Protein-protein interaction of network 9.
