# Supplementary material for: Genetic variations and recurrence in stage III Korean colorectal cancer: Insights from tumor-only mutation analysis
Source: PLoS One. 2025 May 23;20(5):e0323302. doi: 10.1371/journal.pone.0323302 (PMC12101642; doi:10.1371/journal.pone.0323302)
Supplement: S4 File — (DOCX) [file pone.0323302.s011.docx]

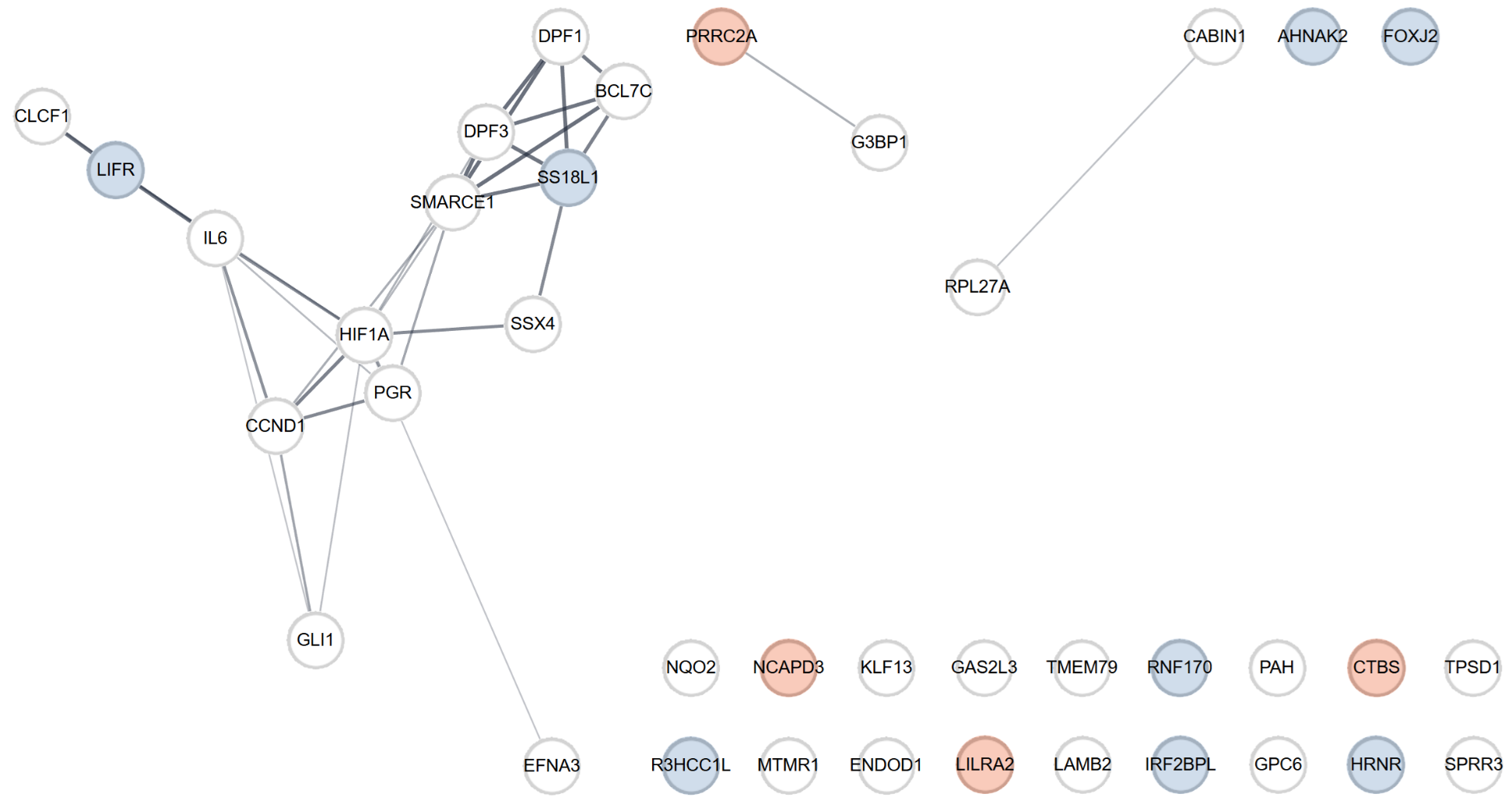


Figure A in S4 File. Protein-protein interaction of network 5.


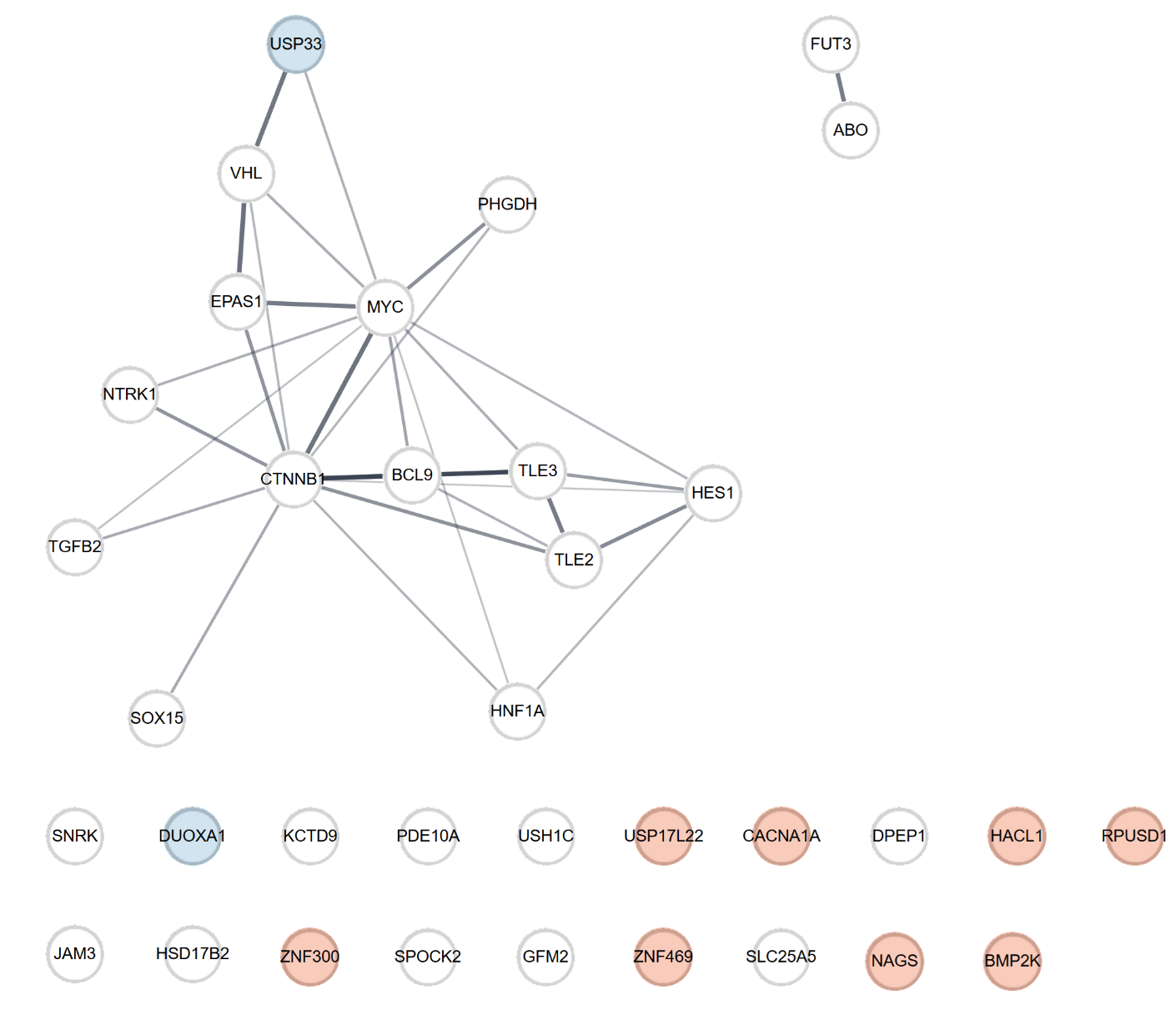


Figure B in S4 File. Protein-protein interaction of network 8.
