# Supplementary figures and images for: Genetic variations and recurrence in stage III Korean colorectal cancer: Insights from tumor-only mutation analysis
Source: PLoS One. 2025 May 23;20(5):e0323302. doi: 10.1371/journal.pone.0323302 (PMC12101642; doi:10.1371/journal.pone.0323302)

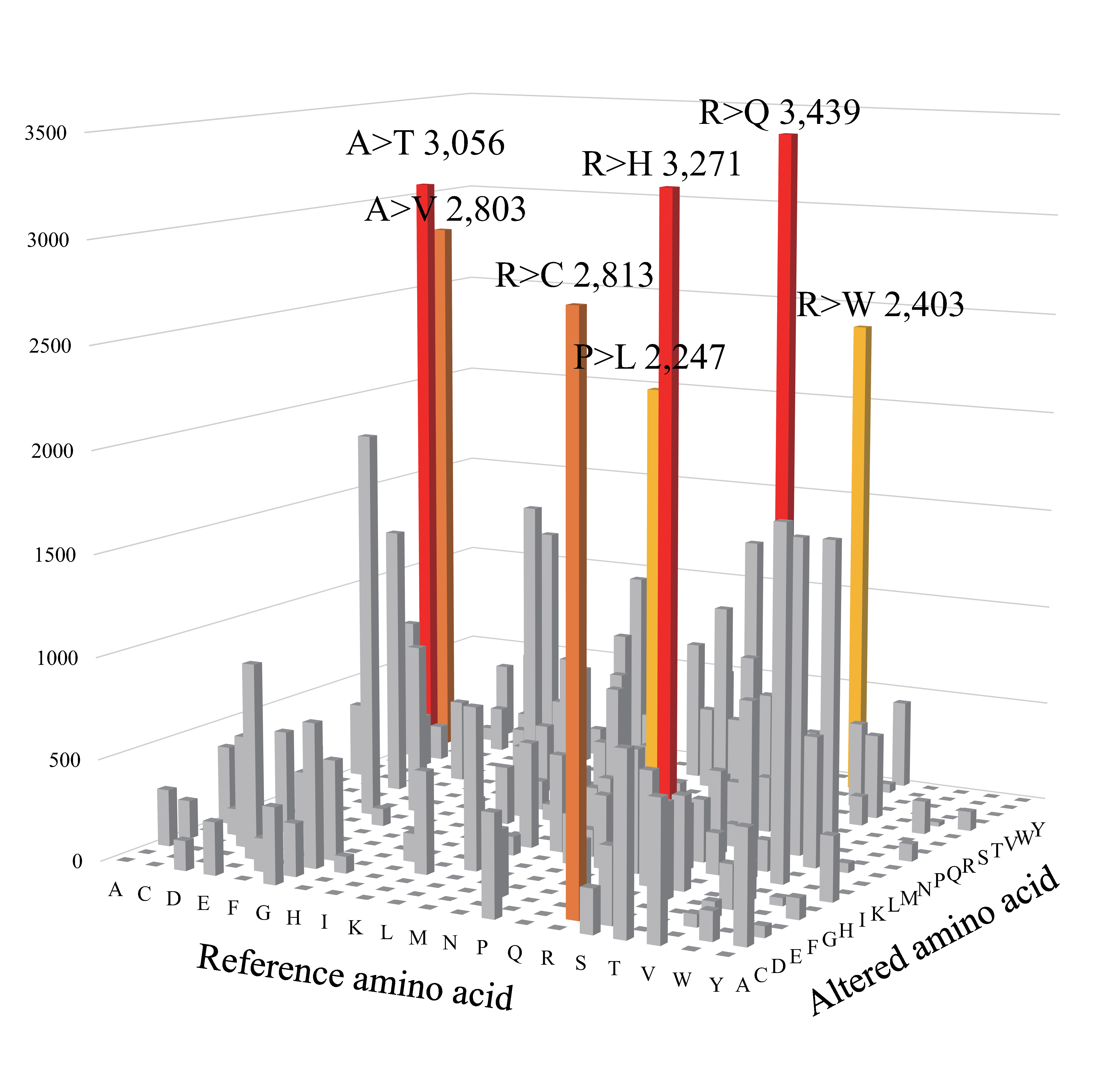

Supplement: S1 Figure — (TIF) [file pone.0323302.s012.tif]
