# Supplementary material for: Genetic variations and recurrence in stage III Korean colorectal cancer: Insights from tumor-only mutation analysis
Source: PLoS One. 2025 May 23;20(5):e0323302. doi: 10.1371/journal.pone.0323302 (PMC12101642; doi:10.1371/journal.pone.0323302)
Supplement: Attachment — Submitted filename: response_to_reviewers.docx [file pone.0323302.s014.docx]

To the Editor and Reviewers
*PLOS ONE*

March 28, 2025

**Subject**: Response to Decision Letter - PLOS ONE Decision: Revision required [PONE-D-24-54119] -[EMID:08656a48aa6d79c6]

We would like to express our sincere gratitude to the editor and reviewers for their thorough and constructive feedback on our manuscript. We have carefully considered all comments and have revised the manuscript accordingly. Below, we provide a detailed point-by-point response to each comment, outlining the corresponding revisions and justifications.

For clarity, our responses are highlighted in blue, while the reviewers’ original comments are shown in black. We hope that our revisions and responses satisfactorily address all concerns, and we welcome any further feedback.

Thank you for your time and consideration.

Sincerely,

The Authors of PONE-D-24-54119

BELOW ARE OUR RESPONSES TO THE EDITOR’S AND REVIEWERS’ COMMENTS.

OUR RESPONSES ARE HIGHLIGHTED IN BLUE.

PONE-D-24-54119

Genetic Variations and Recurrence in Stage III Korean Colorectal Cancer: Insights from Tumor-Only Mutation Analysis

PLOS ONE

Dear Dr.  Yang,
Thank you for submitting your manuscript to PLOS ONE. After careful consideration, we feel that it has merit but does not fully meet PLOS ONE’s publication criteria as it currently stands. Therefore, we invite you to submit a revised version of the manuscript that addresses the points raised during the review process.

Please submit your revised manuscript by Mar 28 2025 11:59PM. If you will need more time than this to complete your revisions, please reply to this message or contact the journal office at [plosone@plos.org](mailto:plosone@plos.org). When you're ready to submit your revision, log on to <https://www.editorialmanager.com/pone/> and select the 'Submissions Needing Revision' folder to locate your manuscript file.

Please include the following items when submitting your revised manuscript:

- A rebuttal letter that responds to each point raised by the academic editor and reviewer(s). You should upload this letter as a separate file labeled 'Response to Reviewers'.
  - We have prepared a detailed, point-by-point rebuttal letter in response to all comments raised by the academic editor and reviewers. This document has been uploaded as a separate file labeled "Response to Reviewers".
- A marked-up copy of your manuscript that highlights changes made to the original version. You should upload this as a separate file labeled 'Revised Manuscript with Track Changes'.
  - A marked-up version of the revised manuscript, showing all changes made to the original version, has been uploaded as "Revised Manuscript with Track Changes." All modifications are indicated in red font, and the Microsoft Word track changes feature has also been used.
- An unmarked version of your revised paper without tracked changes. You should upload this as a separate file labeled 'Manuscript'.
  - An unmarked version of the revised manuscript, without tracked changes, has been uploaded as "Manuscript".

If you would like to make changes to your financial disclosure, please include your updated statement in your cover letter. Guidelines for resubmitting your figure files are available below the reviewer comments at the end of this letter.

If applicable, we recommend that you deposit your laboratory protocols in protocols.io to enhance the reproducibility of your results. Protocols.io assigns your protocol its own identifier (DOI) so that it can be cited independently in the future. For instructions see: [https://journals.plos.org/plosone/s/submission-guidelines#loc-laboratory-protocols](https://track.editorialmanager.com/CL0/https:%2F%2Fjournals.plos.org%2Fplosone%2Fs%2Fsubmission-guidelines%23loc-laboratory-protocols/1/010f0194f63cd843-13d5630a-d94a-482a-b566-88d431390d4d-000000/-cO9iP0SLeEFDA4GmO06xORGrDLcFy-hcph0R9Fy1mU=197). Additionally, PLOS ONE offers an option for publishing peer-reviewed Lab Protocol articles, which describe protocols hosted on protocols.io. Read more information on sharing protocols at [https://plos.org/protocols?utm_medium=editorial-email&utm_source=authorletters&utm_campaign=protocols](https://track.editorialmanager.com/CL0/https:%2F%2Fplos.org%2Fprotocols%3Futm_medium=editorial-email%26utm_source=authorletters%26utm_campaign=protocols/1/010f0194f63cd843-13d5630a-d94a-482a-b566-88d431390d4d-000000/q4BegUZSYP1z5qbm6ej3a71jtFyEMdMTaN_BT2vx6xM=197).

We look forward to receiving your revised manuscript.

Kind regards,

Nejat Mahdieh
Academic Editor
PLOS ONE

Journal Requirements:

When submitting your revision, we need you to address these additional requirements.

1.Please ensure that your manuscript meets PLOS ONE's style requirements, including those for file naming. The PLOS ONE style templates can be found at

<https://journals.plos.org/plosone/s/file?id=wjVg/PLOSOne_formatting_sample_main_body.pdf> and

[https://journals.plos.org/plosone/s/file?id=ba62/PLOSOne_formatting_sample_title_authors_affiliations.pdf](https://track.editorialmanager.com/CL0/https:%2F%2Fjournals.plos.org%2Fplosone%2Fs%2Ffile%3Fid=ba62%2FPLOSOne_formatting_sample_title_authors_affiliations.pdf/1/010f0194f63cd843-13d5630a-d94a-482a-b566-88d431390d4d-000000/cbOdUInGiZq1Gv_Rad2CoqpkaRBi_zVLfBpMWEdBlYg=197)

We have thoroughly rechecked our manuscript to ensure compliance with PLOS ONE’s style guidelines. In particular, we have corrected file names to match the journal’s conventions (e.g., “Response to Reviewers,” “Revised Manuscript with Track Changes,” “Manuscript”). We apologize for any previous inconsistencies and have now resolved these issues.

2. Thank you for uploading your study's underlying data set. Unfortunately, the repository you have noted in your Data Availability statement does not qualify as an acceptable data repository according to PLOS's standards.

At this time, please upload the minimal data set necessary to replicate your study's findings to a stable, public repository (such as figshare or Dryad) and provide us with the relevant URLs, DOIs, or accession numbers that may be used to access these data. For a list of recommended repositories and additional information on PLOS standards for data deposition, please see <https://journals.plos.org/plosone/s/recommended-repositories>.

We acknowledge that our initial repository (KoNA) does not meet PLOS ONE’s standards. To address this, we have revised our Data Availability Statement to clarify that our dataset is currently under restricted access due to national research program requirements because of legal and ethical restrictions. However, it will be publicly released through the Korean BioData Station (K-BDS; https://kbds.re.kr/) under the accession IDs KAP220472, KAP220473, and KAP230611. Researchers may request early access via the K-BDS platform according to applicable guidelines.

Accordingly, the original Data Availability Statement:

*“The raw data were deposited in the Korean Nucleotide Archive (KoNA) under accession IDs KAP220472, KAP220473 and KAP230611.”*

has been revised to the following:

*“Data cannot be shared publicly because of legal and ethical restrictions related to the national research program under which the data were generated. The data are scheduled for public release through the Korean BioData Station (https://kbds.re.kr/) under accession numbers KAP220472, KAP220473, and KAP230611. Until public release, access may be requested via the K-BDS platform. We respectfully request an exemption from the open data policy until the data are released according to this schedule”*

We hope this revised statement meets the journal’s requirements and provides a clear path for data access and future availability.

[Note: HTML markup is below. Please do not edit.]

Reviewers' comments:

Reviewer's Responses to Questions

**Comments to the Author**

1. Is the manuscript technically sound, and do the data support the conclusions?

The manuscript must describe a technically sound piece of scientific research with data that supports the conclusions. Experiments must have been conducted rigorously, with appropriate controls, replication, and sample sizes. The conclusions must be drawn appropriately based on the data presented.

Reviewer #1: Partly

Reviewer #2: Yes

2. Has the statistical analysis been performed appropriately and rigorously?

Reviewer #1: Yes

Reviewer #2: Yes

3. Have the authors made all data underlying the findings in their manuscript fully available?

The [PLOS Data policy](http://track.editorialmanager.com/CL0/http:%2F%2Fwww.plosone.org%2Fstatic%2Fpolicies.action%23sharing/1/010f0194f63cd843-13d5630a-d94a-482a-b566-88d431390d4d-000000/9NDraQObGoxUPpeyw2aLyIorxWoKDLNbJVprfMnWnds=197) requires authors to make all data underlying the findings described in their manuscript fully available without restriction, with rare exception (please refer to the Data Availability Statement in the manuscript PDF file). The data should be provided as part of the manuscript or its supporting information, or deposited to a public repository. For example, in addition to summary statistics, the data points behind means, medians and variance measures should be available. If there are restrictions on publicly sharing data—e.g. participant privacy or use of data from a third party—those must be specified.

Reviewer #1: Yes

Reviewer #2: Yes

4. Is the manuscript presented in an intelligible fashion and written in standard English?

PLOS ONE does not copyedit accepted manuscripts, so the language in submitted articles must be clear, correct, and unambiguous. Any typographical or grammatical errors should be corrected at revision, so please note any specific errors here.

Reviewer #1: Yes

Reviewer #2: Yes

5. Review Comments to the Author

Please use the space provided to explain your answers to the questions above. You may also include additional comments for the author, including concerns about dual publication, research ethics, or publication ethics. (Please upload your review as an attachment if it exceeds 20,000 characters)

Reviewer #1: Dear Editor,
I appreciate the opportunity to review the manuscript PONE-D-24-54119, titled "Genetic Variations and Recurrence in Stage III Korean Colorectal Cancer: Insights from Tumor-Only Mutation Analysis." This study addresses a critical clinical question regarding the genetic factors contributing to colorectal cancer (CRC) recurrence in stage III patients. The authors have employed whole-exome sequencing (WES) and machine learning models to identify potential genetic markers of recurrence, making a valuable contribution to precision oncology. The study is well-designed and has several major strengths that enhance its scientific and clinical impact. However, I have the following major concerns:
1.The study utilizes tumor-only sequencing, which does not differentiate between somatic and germline mutations. This may result in the misclassification of inherited variants as tumor-specific, potentially affecting clinical interpretations.

We appreciate this important observation. Although we applied strict filtering using multiple population databases (gnomAD, ExAC, 1000 Genomes, etc.) to reduce germline contamination, we acknowledge that some germline variants may remain. In the revised manuscript, we have: Conducted an additional variant allele frequency (VAF) analysis to estimate the likelihood of each somatic single nucleotide mutation (SSM) being germline in origin. Determined that approximately 100 out of 221 SSMs had a >50% probability of being germline. Added explicit cautions in the Introduction, Methods, Results, and Discussion to highlight this limitation. Included a new Supplementary Table S7 summarizing these findings.

2.The study focuses exclusively on Korean patients with stage III colorectal cancer. Given that genetic and environmental factors influencing recurrence may differ across ethnic groups and CRC stages, the applicability of these findings to broader populations is limited. Please discuss this limitation.

We agree that restricting the cohort to Korean stage III CRC patients may limit generalizability to other ethnicities or CRC stages. However, Korea has a relatively homogeneous population, and a significant proportion of CRC-related deaths occur in stage III patients here. We have clarified in the Introduction that our aim was to address a critical issue in this specific national context, but we also acknowledge the importance of validating these findings in more diverse populations.

3.While the study identifies statistically significant mutations, it does not confirm their functional impact through experimental validation (e.g., gene expression studies, protein function assays). Without biological validation, the clinical relevance of these mutations remains uncertain.

We concur that experimental validation (e.g., gene expression or protein function assays) would strengthen our findings. While new experiments were not feasible within this study’s scope, we performed a limited in silico validation by examining available public datasets for key genes (e.g., REXO1). We found indications (e.g., correlation with ARID3A) suggesting a functional role, but emphasize that further biological confirmation is necessary. We have added a paragraph in the Discussion regarding this limitation.

4.The CRC recurrence prediction model, developed using machine learning (PyCaret), achieved an AUC of 0.77. While this suggests moderate predictive performance, further validation with external patient cohorts is necessary before clinical application.

We agree. Due to the lack of publicly available tumor-only recurrence datasets, we could not perform external validation. We have acknowledged this shortcoming in the Discussion and highlighted the need for independent cohorts. As more datasets become available, we plan to refine and validate our model.

5.The study considers clinical variables such as T-category, N-category, and preoperative carcinoembryonic antigen (CEA) levels but does not account for lifestyle factors, treatment adherence, or comorbidities, which may also influence CRC recurrence. Including such factors would provide a more comprehensive analysis.

We appreciate this suggestion. Our dataset focused primarily on molecular and core clinical variables from medical records, and data on lifestyle or comorbidities were not available. We have acknowledged this limitation in the Discussion, citing relevant research that underscores the importance of such factors. We recommend that future studies incorporate more comprehensive clinical variables.

6.The study excludes tumors with >24 mutations per megabase to remove hypermutated samples. However, hypermutation status plays a crucial role in immune response and tumor evolution. Its exclusion may limit insights into the mechanisms of CRC recurrence.
I appreciate the authors' efforts and believe addressing these concerns will further strengthen the study.

We initially excluded 13 hypermutated cases (5 recurrence, 8 non-recurrence) for consistency in our main analysis. To address this concern, we performed a supplementary analysis on these 13 samples (see Supplementary File S1, Tables C–D and Figures E–H). These results are also summarized in the revised Methods, Results, and Discussion. We acknowledge the potential importance of hypermutated tumors and recommend future studies with larger hypermutated cohorts.

Reviewer #2: The study provides valuable insights into the genetics of CRC recurrence; however, it would benefit from functional validation, external cohort validation, and broader population studies. I recommend considering minor revisions to enhance methodological robustness and clinical applicability. Below are my comments:
•While the study filters common variants using gnomAD, ExAC, dbSNP, 1000 Genomes Project, KOVA2, and KRGDB, how do the authors address database inaccuracies or population-specific variants that may lead to false positives (wrongly retained somatic variants) or false negatives (important tumor mutations mistakenly removed)?

To minimize false positives, we used the union of multiple databases (gnomAD, ExAC, dbSNP, 1000 Genomes, KOVA2, KRGDB). We acknowledge that some true mutations might be filtered out (false negatives), but this approach increases confidence that retained mutations are truly somatic. We have clarified this rationale in the revised manuscript.

•WES cannot reliably detect large structural variations, copy number alterations (CNVs), or epigenetic modifications, which may be critical in CRC recurrence. Could the authors provide explanations or alternative approaches to account for these limitations?

We agree WES has limited capacity for detecting structural variations, CNVs, or epigenetic modifications. While tools like ECOLE exist to estimate CNVs from WES, their accuracy remains insufficient for clinical use. We highlight these limitations in the revised Discussion and emphasize the need for expanded genomic profiling methods.

•The study utilizes Ingenuity Pathway Analysis (IPA) for protein-protein interaction (PPI) network analysis, which relies on pre-existing knowledge. However, novel mutations and unknown interactions may be overlooked without experimental validation (e.g., CRISPR functional studies, RNA-seq for expression analysis). Could the authors discuss the potential impact of this limitation?

IPA draws on a curated database, potentially missing novel interactions. To partially address this, we cross-referenced results with the STRING database. Notably, *SS18L1* (associated with non-recurrence) formed more connections in STRING than in IPA, suggesting further experimental study is warranted. We discuss these points in the revised Discussion and include STRING-based network visualizations in Supplementary File S4.

•The study identifies seven splicing site mutations and predicts their effects using SpliceAI. However, without RNA sequencing (RNA-seq) validation, it remains unclear whether these mutations actually result in abnormal splicing events in CRC patients. How do the authors address this concern?

We acknowledge that RNA-seq validation is the gold standard to confirm splicing defects. Due to data and IRB constraints, we could not conduct such experiments. As an alternative, we used SpliceAPP alongside SpliceAI; both tools independently supported potential splicing effects for mutations in *LILRA2* and *SRPRA*, though they differed on *SERPINB6*. These findings are included in the revised Results and Supplementary Table S5.

•An important limitation of this study is its exclusive focus on somatic mutations in tumor samples, without assessing germline risk variants that may contribute to hereditary CRC syndromes (e.g., Lynch syndrome, APC mutations in FAP). Acknowledging germline contributions could enhance risk stratification and patient management strategies. Could the authors discuss how future studies might address this gap?

Germline risk variants (e.g., Lynch syndrome, FAP) are crucial but could not be systematically assessed without matched normal samples. We now explicitly state this limitation in the Discussion, noting that future tumor-normal paired studies are needed for comprehensive hereditary risk analysis.

6. PLOS authors have the option to publish the peer review history of their article ([what does this mean?](https://track.editorialmanager.com/CL0/https:%2F%2Fjournals.plos.org%2Fplosone%2Fs%2Feditorial-and-peer-review-process%23loc-peer-review-history/1/010f0194f63cd843-13d5630a-d94a-482a-b566-88d431390d4d-000000/0FMGc9QiYTxTs5E-iu1A6g5MZ7okFUiCfaxyC-azCRQ=197)). If published, this will include your full peer review and any attached files.


If you choose “no”, your identity will remain anonymous but your review may still be made public.


**Do you want your identity to be public for this peer review?** For information about this choice, including consent withdrawal, please see our [Privacy Policy](https://track.editorialmanager.com/CL0/https:%2F%2Fwww.plos.org%2Fprivacy-policy/1/010f0194f63cd843-13d5630a-d94a-482a-b566-88d431390d4d-000000/mS8BItlnMUMpaJUGlL6Gsozi2zUgV0i0UrRSedRCaf0=197).

Reviewer #1: **Yes:** Amirhassan Rabbani

Reviewer #2: No

[NOTE: If reviewer comments were submitted as an attachment file, they will be attached to this email and accessible via the submission site. Please log into your account, locate the manuscript record, and check for the action link "View Attachments". If this link does not appear, there are no attachment files.]

While revising your submission, please upload your figure files to the Preflight Analysis and Conversion Engine (PACE) digital diagnostic tool, [https://pacev2.apexcovantage.com/](https://track.editorialmanager.com/CL0/https:%2F%2Fpacev2.apexcovantage.com%2F/1/010f0194f63cd843-13d5630a-d94a-482a-b566-88d431390d4d-000000/dKB3uyWbBjil0l_VTFCUeLkcZ2kU7RJunkDM-i4juLg=197). PACE helps ensure that figures meet PLOS requirements. To use PACE, you must first register as a user. Registration is free. Then, login and navigate to the UPLOAD tab, where you will find detailed instructions on how to use the tool. If you encounter any issues or have any questions when using PACE, please email PLOS at [figures@plos.org](mailto:figures@plos.org). Please note that Supporting Information files do not need this step.

Thank you for the guidance. We have uploaded all figures included in the manuscript to the PACE system and used the processed output files in the submission, in accordance with PLOS figure requirements.
